# Resistive switching memristors: structures, materials, fabrication techniques, and process challenges for VLSI integration

**DOI:** 10.1039/d6ra05168e

**Published:** 2026-07-29

**Authors:** Robin Singla, Divya Pant, Priyanshu Nautiyal, Anjali Painuly

**Affiliations:** a Department of Electronics and Communication Engineering, Thapar Institute of Engineering and Technology Patiala 147004 Punjab India robinsingla.eng@gmail.com

## Abstract

Memristors have gained popularity in being used as nanodevices to be used as nonvolatile memory devices, neuromorphic computing systems, and even compute-in-memory systems due to their property of nonvolatile resistive switching, power savings, scalability, and simple metal–insulator–metal structures. These memristive switching operations can occur due to different phenomena like oxygen vacancy movement, electrochemical metallization, valence change operations, phase change, and ferroelectric polarization operations. Performance of such devices greatly depends on materials and processing, hence knowing their relationship is very important. This review presents an extensive discussion about various memristor structures, materials used, fabrication processes, testing procedures, and the problems encountered in VLSI scaling down. Special attention has been paid to the switching phenomenon, defect management, interfaces, the movement of conductive filaments, and fabrication variability that considerably influence the performance, endurance, and reliability of memristors. In addition, the importance of the processes used for fabricating memristor devices such as photolithography, chemical vapor deposition, sputtering, electron-beam deposition, and atomic layer deposition has been highlighted. Through the use of improvements in materials science, device architectures, fabrication processes, and system integration techniques, this review outlines the potentials and open issues involved in memristor research. The findings outlined in the discussion offer a basis for furthering the progress of reliable and scalable memristor technology compatible with CMOS circuits.

## Abstract 2

Memristors are widely used as nonvolatile memory devices, neuromorphic computing systems, and compute-in-memory systems due to their nonvolatile resistive switching, power efficiency, scalability, and simple structures.

## Graphical abstract

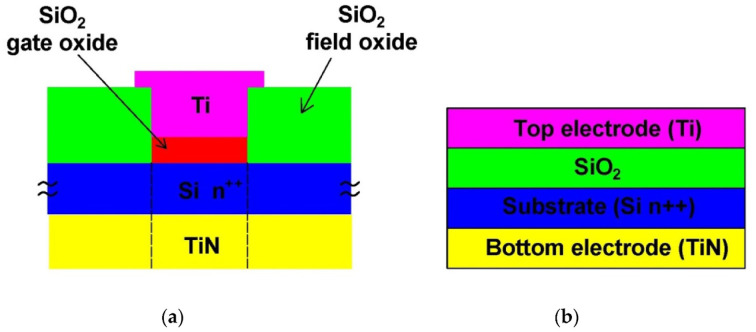

## 1 Introduction

The memristor (memory resistor) is regarded as the fourth fundamental passive circuit element alongside the resistor, capacitor, and inductor. First proposed theoretically by Chua in 1971, the memristor establishes a relationship between electric charge and magnetic flux linkage, where the resistance depends on the history of the applied electrical signal.^1^ Unlike conventional passive devices, memristors can retain their resistance state even after the removal of external power, making them attractive for non-volatile memory applications.

The experimental realization of TiO_2_-based memristive devices by Strukov *et al.* validated this theoretical concept and demonstrated the characteristic pinched hysteresis loop in current–voltage (*I*–*V*) characteristics.^2^ Since then, memristive devices have attracted significant research interest because of their simple two-terminal structure, nanoscale dimensions, low power consumption, and excellent scalability.^3,4^ These properties make memristors promising candidates for next-generation electronic systems including resistive random-access memory (RRAM), neuromorphic computing, and in-memory computing architectures.^5–7^

The switching behavior of memristive devices arises from several physical mechanisms depending on the material system and device configuration. In oxide-based devices, resistive switching is commonly associated with oxygen vacancy migration, conductive filament formation, electrochemical metallization, and valence change mechanisms within the active layer.^8–10^ Different material systems including transition metal oxides, chalcogenides, polymers, and two-dimensional materials have been investigated because of their distinct switching characteristics and defect chemistry.^11–13^ These mechanisms strongly influence switching voltage, endurance, retention, and device reliability.

Different device structures like MIM (Metal Insulator Metal), MIS (Metal Insulator Semiconductor) and crossbar have also been explored to enhance the electrical characteristics as well as the packing density of memristors.^14,15^ Additionally, methods like sputtering, chemical vapor deposition, atomic layer deposition and compatible CMOS processing are vital in deciding the quality of interfaces, the concentration of defects, and the switching reliability.^16,17^

Despite many studies showing that memristors have promising applications, there are still some obstacles in implementing memristors in large-scale VLSI devices. These obstacles include device variability, conductive filament instability, endurance limitations, thermal constraints, and difficulties in integrating with complementary metal oxide semiconductor technologies.^18–20^ It is thus crucial to have a deeper understanding of the interaction among materials, manufacturing processes, switching mechanisms, and electrical properties of the devices.

In this paper, a comprehensive analysis of the memristor device architectures, material technologies, processing methods, electrical measurements, and processing issues pertaining to its VLSI integration is carried out. The main focus is on analyzing the role played by the defect physics, processing conditions, and switching mechanisms in determining the device reliability and scalability issues.

Though many review papers have been written about the materials used for memristors, their switching mechanism, memory architectures, or neuromorphic implementations individually, a complete analysis about the impact of these variables together is missing. This review attempts to fill this knowledge gap by offering a holistic overview of various issues such as devices, materials, processing techniques, electrical properties, and integration in VLSI circuits.

## 2 Structure and switching techniques of memristive devices

The performance of a memristor is heavily influenced by the architecture of the device, the materials used, and the mechanism for switching. Memristive devices can be divided into six main groups based on their structural configuration and active switching layer: Metal–Insulator–Metal (MIM), Metal–Insulator–Semiconductor (MIS), Metal–SiO_2_–Metal (MOM), Metal–Insulator–Semiconductor–Metal (MISM), phase-change memristors, and ferroelectric memristors. These structures can also be used in planar, vertical, or crossbar designs to integrate high-density memory.

### 2.1 Metal–insulator–metal (MIM) structure

**Fig. 1:**
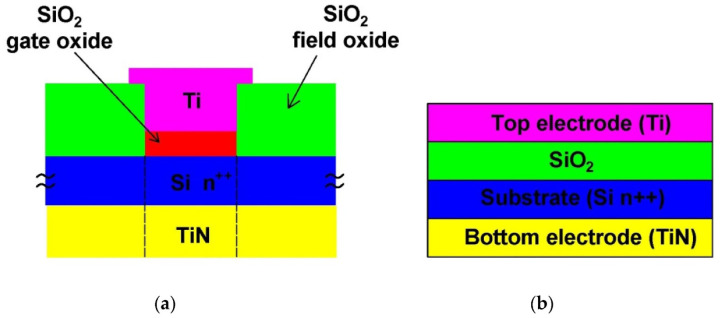
(a) Cross-sectional view of the Ti/SiO_2_/Si(n^++^)/TiN memristor. (b) Schematic illustration of the Metal–Insulator–Metal (MIM) structure, reproduced from ref. 21, G. L. Priya, N. Rawat, A. Sanagavarapu, M. Venkatesh and A. A. Roobert, *Micromachines*, 2023, **14**, 232, licensed under CC BY 4.0, copyright © 2023.

The Metal–Insulator–Metal (MIM) structure is the most widely investigated memristor architecture because of its simple geometry, ease of fabrication, and suitability for nanoscale integration. A typical MIM device consists of an insulating switching layer positioned between two metallic electrodes, where the active layer governs the resistive switching behavior. Fig. 1 illustrates a hybrid silicon substrate-based MIM memristor integrated with a FinFET configuration, as reported by Priya *et al.*^21^

In the reported device, titanium (Ti) functions as the top electrode, titanium nitride (TiN) serves as the bottom electrode, and silicon dioxide (SiO_2_) acts as the insulating switching layer. The device is fabricated on an n++ silicon substrate, providing compatibility with conventional CMOS technology and making the structure attractive for integrated memory applications.

Fig. 1(a) presents the cross-sectional view of the Ti/SiO_2_/Si(n++)/TiN memristor integrated with a FinFET platform. The image highlights the silicon substrate, oxide switching layer, and electrode arrangement used to achieve resistive switching. In contrast, Fig. 1(b) provides a simplified schematic representation of the MIM configuration consisting of a top electrode, insulating layer, and bottom electrode. Together, these figures demonstrate how the electric field is mainly confined within the insulating layer and emphasize the role of interface quality and layer thickness in determining device operation.^21^

The insulating layer plays the most critical role because its defect concentration, thickness, and interface characteristics directly influence switching voltage, resistance ratio, endurance, and device stability. When an external voltage is applied, oxygen vacancies or metal ions migrate inside the dielectric layer, resulting in the formation or rupture of conductive filaments. This process enables reversible switching between the high-resistance state (HRS) and low-resistance state (LRS). Depending on the electrode material and switching layer composition, MIM devices may operate through valence change mechanisms (VCM) or electrochemical metallization (ECM) processes.^9,10^

The FinFET-integrated structure shown in Fig. 1 is particularly important because it demonstrates the possibility of combining memristive switching with existing transistor technology.Such hybrid configurations improve memory density and enable better control of read and write operations, making them attractive for embedded memory and compute-in-memory architectures.^6,14,21^

Compared with more complex architectures, MIM devices offer several advantages including simple fabrication, scalability, and compatibility with high-density crossbar arrays. Their compact two-terminal geometry supports large-scale integration and low-area memory implementation. However, MIM devices also face several challenges. Since switching often depends on stochastic conductive filament formation, variations in oxide thickness, defect concentration, or fabrication conditions can lead to cycle-to-cycle fluctuation and device-to-device variability.^9^ Therefore, precise control of defect engineering and interface optimization remains essential for achieving reliable and reproducible switching performance in future VLSI-integrated memristor systems.

### 2.2 Metal–insulator–semiconductor (MIS) structure

Two-dimensional (2D) material-based memristors have attracted considerable attention because of their atomically thin structure, excellent electrostatic control, and tunable interface properties. Unlike conventional oxide-based MIM devices, where switching is usually governed by conductive filament formation, many MIS memristors operate through modulation of the Schottky barrier formed at the metal–semiconductor interface. This switching mechanism provides better control of carrier transport and often results in improved switching uniformity and reduced variability.

**Fig. 2:**
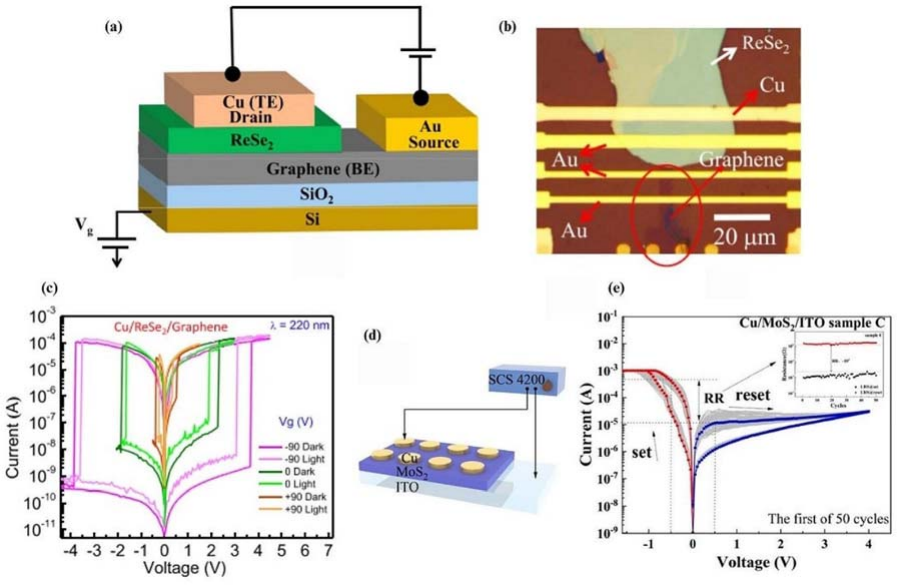
Memristive devices utilizing Schottky barrier height modulation as a resistive switching mechanism. (a) Schematic diagram of the Cu/ReSe_2_/graphene memristor. (b) Optical micrograph of the fabricated device. (c) *I*–*V* characteristics of the Cu/ReSe_2_/graphene device under different gate voltages and deep ultraviolet illumination. (d) Schematic representation of the Cu/MoS_2_/ITO device. (e) *I*–*V* characteristics of the MoS_2_ device demonstrating nonvolatile resistive switching behavior, reproduced from ref. 13, J. Panisilvam *et al.*, *Materials Today Electronics*, 2023, **3**, 100017, licensed under CC BY 4.0, copyright © 2024.

Fig. 2(a) illustrates the Cu/ReSe_2_/graphene memristive device, where graphene acts as the bottom electrode and ReSe_2_ functions as the active switching layer. Unlike oxide-based filamentary devices, resistive switching in this configuration mainly originates from Schottky barrier modulation at the metal–semiconductor interface. The barrier height controls carrier injection and transport across the junction, allowing resistance states to be altered without requiring permanent conductive filament formation. The optical image shown in Fig. 2(b) confirms the layered device geometry and proper alignment of the electrodes. The corresponding *I*–*V* characteristics presented in Fig. 2(c) reveal that the switching response strongly depends on gate voltage and deep ultraviolet (DUV) illumination conditions. Under gate bias and optical excitation, the carrier concentration and interface barrier height are modified, resulting in controllable changes in resistance. This behavior demonstrates the strong electrostatic tunability of 2D material-based devices. A similar mechanism is observed in the Cu/MoS_2_/ITO device shown in Fig. 2(d). The *I*–*V* characteristics in Fig. 2(e) exhibit stable and nonvolatile switching behavior over repeated cycles, indicating reliable memory operation.^13^ In such devices, switching is primarily associated with modulation of interfacial carrier transport and defect-assisted charge transfer rather than complete filament growth and rupture. The switching characteristics of MIS memristors differ significantly from conventional filamentary devices. In oxide-based MIM systems, conductive filament formation often produces abrupt resistance transitions and larger cycle-to-cycle variability due to stochastic defect migration. In contrast, Schottky barrier-controlled switching generally produces smoother resistance modulation and stronger dependence on gate voltage, temperature, and interface properties. Experimental signatures such as gradual current modulation, gate-controlled transport, and optical sensitivity therefore help distinguish interface-dominated switching from filamentary conduction mechanisms. Because of their atomically sharp interfaces, tunable band structures, and enhanced electrostatic control, 2D material-based MIS memristors provide improved switching uniformity and higher tunability compared with many oxide-based systems. From a VLSI integration perspective, these devices offer the additional advantage of atomically thin active layers that can potentially support highly scaled memory and computing architectures. However, practical implementation remains challenging because the growth and transfer of high-quality 2D materials must be compatible with conventional CMOS process flows and back-end-of-line (BEOL) thermal constraints. Furthermore, issues such as contamination control, wafer-scale uniformity, interface stability, and reproducible large-area fabrication must be carefully addressed to ensure reliable device operation. Therefore, although 2D material-based MIS memristors demonstrate significant potential for future memory and neuromorphic applications, further advances in CMOS-compatible fabrication and BEOL-compliant integration strategies are required before large-scale industrial deployment can be realized. These characteristics make them promising candidates for neuromorphic hardware, adaptive electronics, and future low-power computing architectures.^13,22^

### 2.3 Metal–SiO_2_–metal (MOM) structure

The Metal–SiO_2_–Metal (MOM) structure is an important class of resistive switching device in which silicon dioxide (SiO_2_) functions as the insulating switching layer between two metallic electrodes. Because SiO_2_ is already a fundamental dielectric material in CMOS technology, MOM devices offer strong compatibility with existing semiconductor manufacturing processes and therefore attract attention for embedded memory and VLSI-integrated applications.

**Fig. 3:**
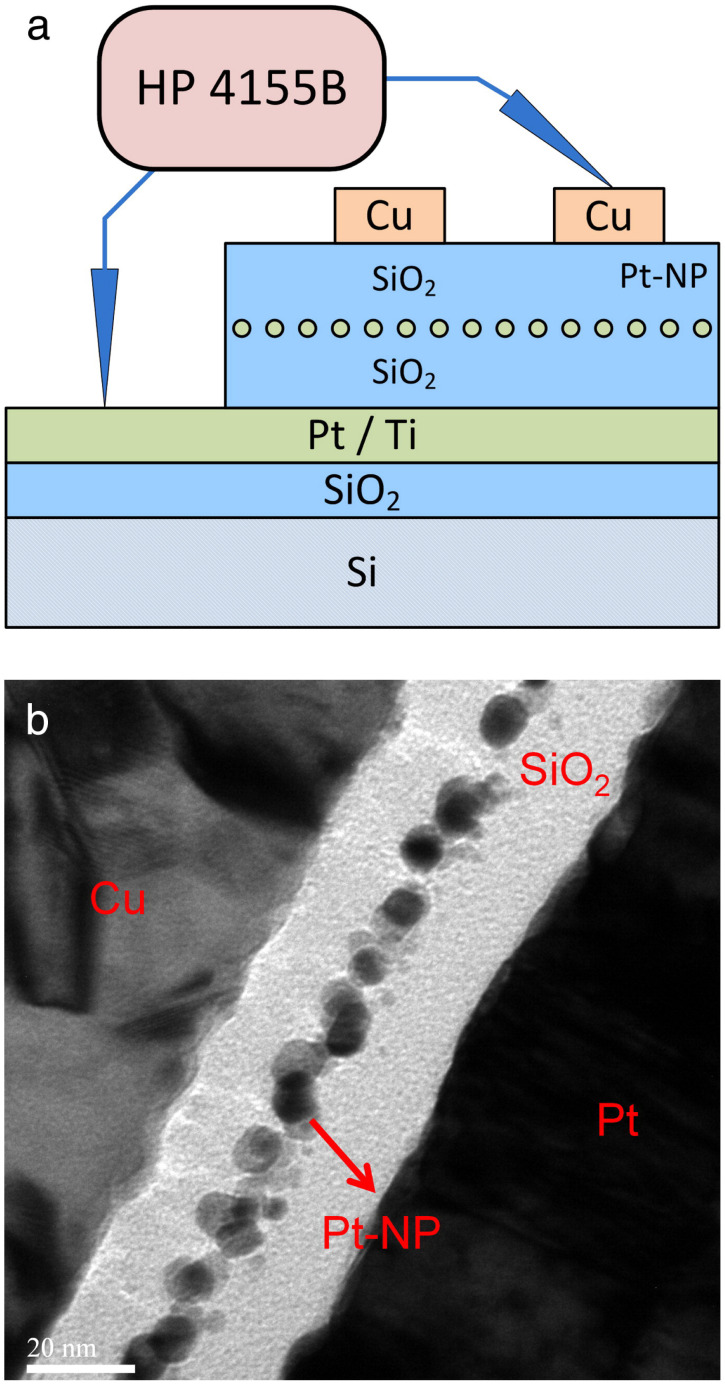
(a) Schematic of the Cu/Pt-nanoparticle (Pt-NP)-embedded SiO_2_/Pt memory structure along with the electrical measurement setup. (b) Cross-sectional HRTEM image showing Pt nanoparticles embedded inside the SiO_2_ layer, reproduced from ref. 23 with permission from Elsevier, C.-Y. Liu, J.-J. Huang and C.-H. Lai, “Resistive switching characteristics of a Pt nanoparticle-embedded SiO_2_-based memory”, *Thin Solid Films*, 2013, **529**, 107–110, copyright 2013.

Fig. 3(a) illustrates the Cu/Pt-nanoparticle-embedded SiO_2_/Pt memristive structure together with the electrical measurement configuration. The device consists of a Cu top electrode, a Pt bottom electrode, and a SiO_2_ switching layer containing intentionally embedded Pt nanoparticles. These nanoparticles are introduced to modify local electric-field distribution and act as preferred sites for defect accumulation and charge trapping. The cross-sectional HRTEM image shown in Fig. 3(b) confirms the uniform distribution of Pt nanoparticles within the SiO_2_ layer. Their controlled placement is particularly important because nanoparticle-induced field enhancement promotes localized defect migration and helps define conductive pathways more reproducibly than in conventional homogeneous oxide layers.^23^ Resistive switching in SiO_2_-based MOM devices is generally associated with defect-assisted transport and soft dielectric breakdown. Under an applied electric field, oxygen-vacancy generation and migration occur inside the insulating layer, leading to the formation of conductive channels that switch the device from the high-resistance state (HRS) to the low-resistance state (LRS). In nanoparticle-assisted devices, the Pt nanoparticles intensify the local electric field and reduce the randomness of filament nucleation, thereby improving switching repeatability and resistance-state stability.^9,23^

Compared with transition-metal oxide memristors such as HfO_2_ or Ta_2_O_5_, SiO_2_-based devices provide superior CMOS compatibility because SiO_2_ processing is already well established in semiconductor fabrication. However, the wide bandgap and high dielectric strength of SiO_2_ generally require larger forming voltages and stronger electric fields to initiate switching. This increases power consumption and may accelerate dielectric degradation during repeated switching cycles. Consequently, reliable MOM operation depends strongly on defect engineering and interface control. Parameters such as nanoparticle concentration, oxide stoichiometry, and electrode diffusion significantly affect filament formation, endurance, and long-term retention. Although nanoparticle-assisted SiO_2_ devices demonstrate improved switching uniformity, controlling defect distribution and minimizing variability remain critical challenges for scalable and CMOS-compatible memristor integration.

### 2.4 Metal–insulator–semiconductor–metal (MISM) structure

The Metal–Insulator–Semiconductor–Metal (MISM) structure represents an extended form of the conventional MIS configuration, where metallic contacts are introduced on both sides of the semiconductor layer. This additional electrode arrangement provides improved control over carrier injection, electric-field distribution, and interfacial transport properties. As a result, MISM devices have attracted attention for applications requiring tunable switching characteristics and enhanced electrical modulation.

**Fig. 4:**
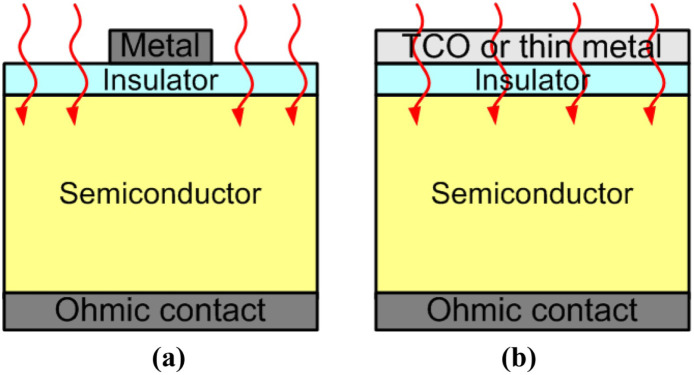
Schematic illustration of metal–insulator–semiconductor structures. (a) Device with an opaque metal electrode where light or carriers enter from the unshadowed region. (b) Device using a transparent conducting oxide (TCO) or thin semi-transparent metal layer, reproduced from ref. 24, C.-H. Lin, F.-H. Ko, C.-C. Huang and Y.-H. Lee, *Sensors*, 2010, **10**, 8797–8826, licensed under CC BY 4.0, copyright © 2010.

Fig. 4(a) illustrates a MISM configuration employing an opaque metallic electrode. In this arrangement, carrier injection or optical excitation occurs through the unshadowed semiconductor region. Fig. 4(b) presents an alternative structure using a transparent conducting oxide (TCO) or thin semi-transparent metallic layer. The use of transparent electrodes improves electric-field penetration and allows more direct interaction between external excitation and the semiconductor layer.^24^ Unlike conventional MIM devices, where switching is generally dominated by conductive filament formation inside the insulating layer, MISM devices often exhibit hybrid switching behavior involving both bulk and interface-controlled processes. Resistive switching may arise from conductive filament formation within the dielectric layer together with charge trapping, interface-state modulation, or barrier-height variation at the insulator–semiconductor interface. Consequently, electrical conduction is influenced not only by defect migration but also by carrier transport across the semiconductor junction. The semiconductor layer therefore plays a critical role in determining device operation. Parameters such as semiconductor doping concentration, carrier mobility, and interface quality directly influence barrier height, charge distribution, and resistance modulation. Because both filamentary and interfacial processes may coexist, MISM devices can demonstrate gradual switching behavior and support multi-level resistance states, which are desirable for analogue memory and neuromorphic applications. The transparent conducting oxide (TCO) or semi-transparent metal structure shown in Fig. 4(b) further enhances device tunability by improving carrier injection and electric-field control. Such configurations provide greater flexibility in engineering switching characteristics and may enable optoelectronic or photoresponsive memristive operation.^24^ Compared with basic MIM architectures, MISM structures provide superior interface engineering capability and improved modulation of carrier transport. However, these advantages are accompanied by increased fabrication complexity. Precise control of semiconductor doping, interface contamination, and layer thickness is essential to maintain switching uniformity and long-term reliability. Therefore, although MISM devices offer promising functionality and multi-level switching capability, achieving reproducible large-scale integration remains an important challenge for future CMOS-compatible memristive systems.

### 2.5 Phase-change memristive structure

Phase-change memristive devices operate through reversible structural transformation of the active material rather than through conductive filament formation or defect migration. In these systems, resistance modulation arises from phase transitions between distinct crystal structures having different electrical conductivities. Because switching is governed by changes in lattice arrangement and electronic structure, phase-change memristors generally exhibit improved resistance stability and reduced stochastic variability compared with many filamentary oxide devices.

**Fig. 5:**
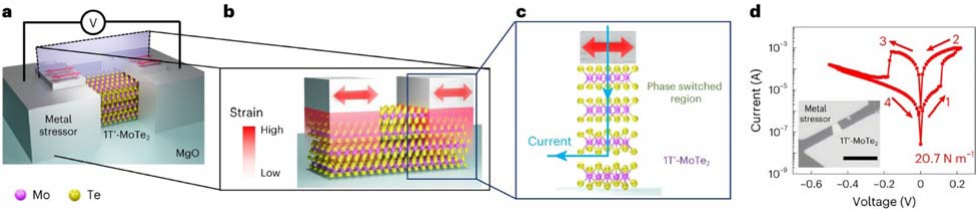
Strain-engineered vertical MoTe_2_ phase-change memristor. (a) Device schematic showing stressed metal contacts inducing strain in 1T′-MoTe_2_. (b) Cross-sectional view illustrating strain distribution. (c) Mechanism of vertical transport through phase-switched MoTe_2_ beneath the contact metal. (d) Typical resistive switching *I*–*V* characteristics of the strain-based memristor (inset: optical micrograph), reproduced from ref 25 with permission from Springer Nature, W. Hou, A. Azizimanesh, A. Dey *et al.*, “Strain engineering of vertical molybdenum ditelluride phase-change memristors”, *Nature Electronics*, 2024, **7**, 8–16, copyright 2024.

Fig. 5(a) illustrates a strain-engineered vertical memristor based on 1T′-MoTe_2_, where stressed metal contacts intentionally generate localized mechanical strain at the metal–semiconductor interface. Unlike conventional phase-change memories that rely primarily on Joule heating, this device employs strain engineering to induce reversible structural transformation within the MoTe_2_ layer. The cross-sectional representation shown in Fig. 5(b) demonstrates that strain is concentrated near the contact region. This localized strain modifies the crystal symmetry and stabilizes different structural phases within the MoTe_2_ layer. As illustrated in Fig. 5(c), electrical conduction occurs through the vertically transported current beneath the contact metal, where the strain-induced phase-switched region governs carrier transport and resistance modulation. The resistive switching characteristics shown in Fig. 5(d) confirm stable resistance modulation associated with reversible phase transformation. In contrast to oxide-based memristors, where switching often depends on random filament nucleation and rupture, phase-change devices rely on controlled lattice reconfiguration.^25^ This distinction reduces switching randomness and provides improved reproducibility. Traditional phase-change materials such as Ge_2_Sb_2_Te_5_ (GST) typically require thermal programming through Joule heating to switch between amorphous and crystalline states. Although such materials provide excellent retention and stable resistance levels, their relatively high programming power and thermal management requirements may complicate large-scale integration. The strain-engineered MoTe_2_ device addresses some of these limitations by replacing thermally driven switching with mechanically induced phase transformation, thereby reducing switching energy and enabling more localized control.^25^ Because phase-transition behavior is strongly influenced by crystal quality, interface stress, and structural uniformity, careful control of material growth and contact engineering remains essential. While phase-change memristors provide superior structural control and reduced variability compared with filamentary devices, maintaining reproducible phase boundaries and ensuring compatibility with CMOS back-end thermal constraints continue to be important challenges for scalable VLSI implementation.

### 2.6 Ferroelectric–memristor unified structure

Ferroelectric memristive devices employ polarization switching within ferroelectric materials to modulate resistance states without relying primarily on conductive filament formation. In these systems, resistance modulation originates from reversible polarization reversal that alters internal electric fields and carrier transport properties. Compared with filament-based oxide memristors, ferroelectric switching generally provides improved uniformity, lower variability, and superior endurance because device operation is governed by domain polarization rather than stochastic filament nucleation.

**Fig. 6:**
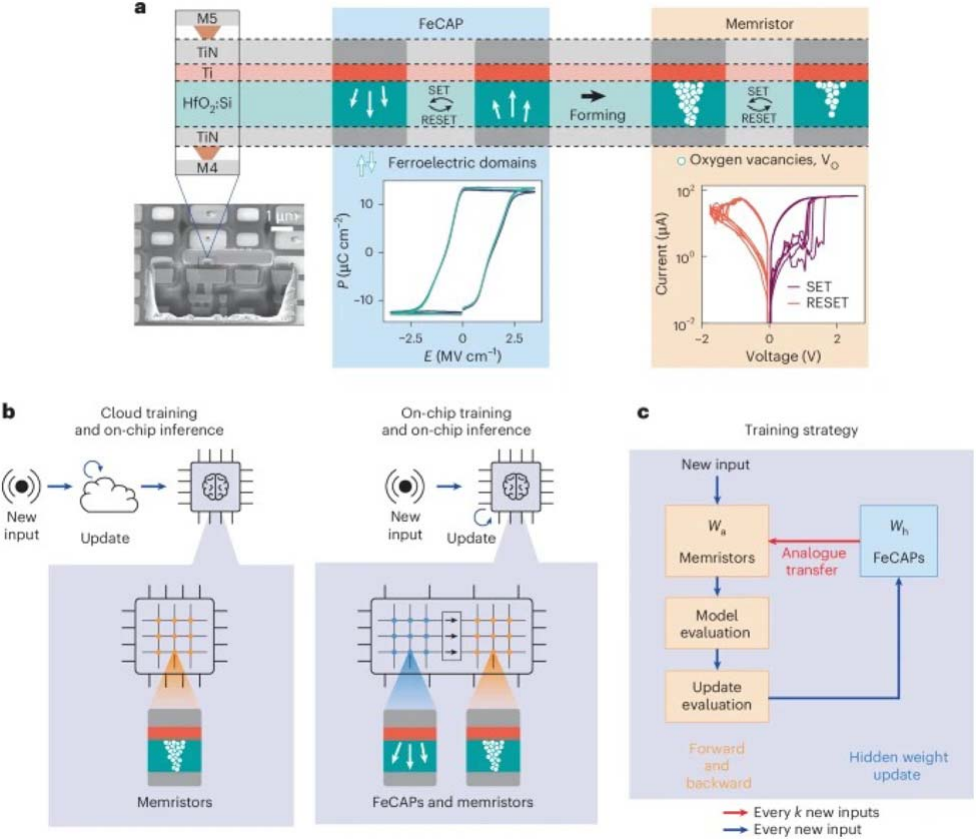
Ferroelectric–memristor unified memory architecture. (a) BEOL-integrated metal–ferroelectric–metal stack that can function either as a FeCAP (showing *P*–*E* hysteresis) or, after forming, as a memristor (butterfly-shaped *I*–*V* curve). (b) Conceptual illustration of off-chip and on-chip training strategies. (c) Proposed training framework leveraging unified ferroelectric–memristor memory, reproduced from ref 26, M. Martemucci, F. Rummens, Y. Malot *et al.*, “A ferroelectric–memristor memory for both training and inference”, *Nature Electronics*, 2025, **8**, 921–933, licensed under CC BY-NC-ND, copyright © 2025.

Fig. 6(a) illustrates a BEOL-integrated metal–ferroelectric–metal stack based on HfO_2_:Si. A significant feature of this architecture is its dual-mode operation. Before forming, the device behaves as a ferroelectric capacitor (FeCAP), exhibiting the characteristic polarization–electric field (*P*–*E*) hysteresis loop associated with ferroelectric domain reversal. After the forming operation, the same physical stack can operate as a memristive device, displaying a butterfly-shaped current–voltage characteristic associated with resistance modulation. In FeCAP mode, switching arises from polarization reversal inside the ferroelectric layer. The reorientation of ferroelectric domains modifies internal electric-field distribution and carrier transport across the device. Unlike filamentary oxide memristors, where conductive paths form and rupture through defect migration, ferroelectric switching is governed primarily by domain dynamics. This mechanism provides smoother resistance modulation, improved cycle-to-cycle reproducibility, and reduced stochastic variability. The use of HfO_2_-based ferroelectrics is particularly important because hafnium oxide is already compatible with advanced CMOS processing and can be integrated within back-end-of-line (BEOL) thermal constraints. Consequently, HfO_2_ ferroelectric systems offer a promising pathway toward scalable and CMOS-compatible non-volatile memory platforms. Fig. 6(b) presents different learning strategies enabled by the unified architecture.

In inference-oriented systems, memristors primarily store analogue synaptic weights used during computation. In contrast, on-chip training requires continuous and high-precision weight updates. To address this challenge, the unified platform separates these tasks: memristors store analogue inference weights, while FeCAP elements retain higher-precision hidden states required for training operations. The proposed training framework summarized in Fig. 6(c) enables efficient analogue weight transfer between FeCAP and memristor modes. This hybrid strategy combines the non-volatility and analogue programmability of memristors with the precision and stability of ferroelectric polarization control.^26^ Compared with conventional filament-based devices, ferroelectric–memristor systems provide several advantages including higher endurance, improved switching uniformity, lower variability, and stronger compatibility with neuromorphic hardware implementations.^26,27^ However, practical deployment still depends on precise control of ferroelectric crystallization, domain stability, and interface quality. Maintaining reliable polarization switching while preserving BEOL compatibility therefore remains a key challenge for large-scale VLSI-integrated ferroelectric memristive systems.

Table 1 presents a comparative analysis of the six memristor structures discussed in this review, highlighting their typical device stacks, dominant switching mechanisms, fabrication approaches, operating voltages, endurance, retention characteristics, scalability, and CMOS integration status. The comparison demonstrates that each memristor architecture offers distinct advantages and limitations depending on the underlying switching mechanism and material system. While filamentary MIM and MOM structures provide high scalability and compatibility with resistive memory applications, MIS and MISM devices offer enhanced interface control and multi-level switching capability. Similarly, phase-change and ferroelectric memristors exhibit improved switching uniformity and endurance, making them promising candidates for emerging neuromorphic and next-generation computing systems.

**Table 1:** Comparative analysis of representative memristor structures and their electrical, fabrication, and integration characteristics compiled from literature reports

Structure	Typical device stack/Active material	Switching mechanism	Fabrication/integration	Operating voltage	Scalability/CMOS status	Typical applications	Key limitations
MIM	TiO_2_, HfO_2_, Ta_2_O_5_ (ref. 2–4)	Filamentary switching (VCM/ECM)^3,10^	Sputtering, ALD, crossbar integration^18^	Moderate	High CMOS compatibility and mature RRAM processing^4^	RRAM, storage memory	Filament instability and switching variability^28,29^
MIS	MoS_2_, ReSe_2_, graphene^13^	Schottky barrier and interface-controlled switching^13^	2D material transfer and semiconductor processing	Low	Promising for low-power integration	Neuromorphic computing	Interface sensitivity and fabrication complexity^30^
MOM (SiO_2_)	Cu/Pt-NP/SiO_2_/Pt^23^	Defect-assisted and soft-breakdown transport^23^	CMOS-compatible oxide deposition	Moderate–High	Excellent CMOS compatibility	Embedded memory	Higher forming requirement and defect sensitivity^23^
MISM	Metal/insulator/semi-conductor/metal^31^	Hybrid interface + filament switching^31^	Multilayer fabrication and interface engineering	Moderate	Supports multi-level operation	Advanced memory and analogue devices	Complex fabrication and interface control^31^
Phase-change	MoTe_2_, GST^25,32^	Structural phase transition	Thermal or strain-assisted switching	Moderate	Suitable for storage-class memory	Storage and phase-change memory	Thermal management and phase stability^25^
Ferroelectric	HfO_2_:Si, FeCAP stacks^26,27^	Polarization switching	BEOL-compatible ferroelectric integration	Low	Excellent CMOS/BEOL compatibility	Neuromorphic and low-power memory	Material optimization and domain variability^26^

Overall, the memristor structures discussed in this section exhibit different strengths and limitations. MIM and MOM devices offer mature fabrication routes and good CMOS compatibility, whereas MIS and MISM structures provide improved control over carrier transport and interface properties. Phase-change and ferroelectric memristors enable enhanced endurance and multilevel operation but introduce additional material and integration challenges. Therefore, the selection of a suitable memristor architecture depends strongly on the target application and system-level requirements.

## 3 Materials used in memristor devices and their characterization

The selection of active materials plays a crucial role in determining the switching behavior and overall performance of memristive devices. Along with device architecture, material composition strongly influences defect migration, charge transport, and resistance modulation mechanisms. Different material systems exhibit distinct electrical properties and defect dynamics, which directly affect important device parameters such as switching voltage, ON/OFF ratio, endurance, retention, and operational stability.

In reported memristive devices, materials are generally classified into three major categories: two-dimensional (2D) materials, metal oxides, and bulk or hybrid material systems. Each category demonstrates unique switching characteristics depending on its crystal structure, defect chemistry, and interface properties. For example, oxide-based memristors commonly rely on oxygen-vacancy migration and filament formation, whereas 2D materials often exhibit interface-controlled or charge-trapping behavior enabled by atomically thin layers and tunable band structures.

The microstructural and chemical properties of the active switching layer further influence device reliability and variability. Parameters such as crystallinity, grain boundaries, stoichiometry, vacancy concentration, and electrode diffusion significantly affect switching uniformity and long-term performance. Therefore, careful material engineering and interface control are essential for developing reliable and scalable memristive systems.

To investigate these material-dependent characteristics, several structural and spectroscopic characterization techniques are widely employed. Scanning electron microscopy (SEM), transmission electron microscopy (TEM), atomic force microscopy (AFM), X-ray diffraction (XRD), and Raman spectroscopy provide valuable information regarding surface morphology, crystal structure, phase transformation, and defect distribution. These characterization methods help establish correlations between nanoscale material properties and the electrical performance of memristive devices.^11,12^

### 3.1 Two-dimensional (2D) materials

Two-dimensional (2D) materials have emerged as promising candidates for next-generation memristive devices because of their atomically thin geometry, tunable electronic properties, and excellent electrostatic control. Unlike conventional bulk oxide materials, 2D systems provide atomically sharp interfaces, reduced short-channel effects, and enhanced flexibility in tailoring charge transport and switching characteristics.

These features enable improved interface engineering and open new possibilities for low-power and highly scalable memristive devices.

**Fig. 7:**
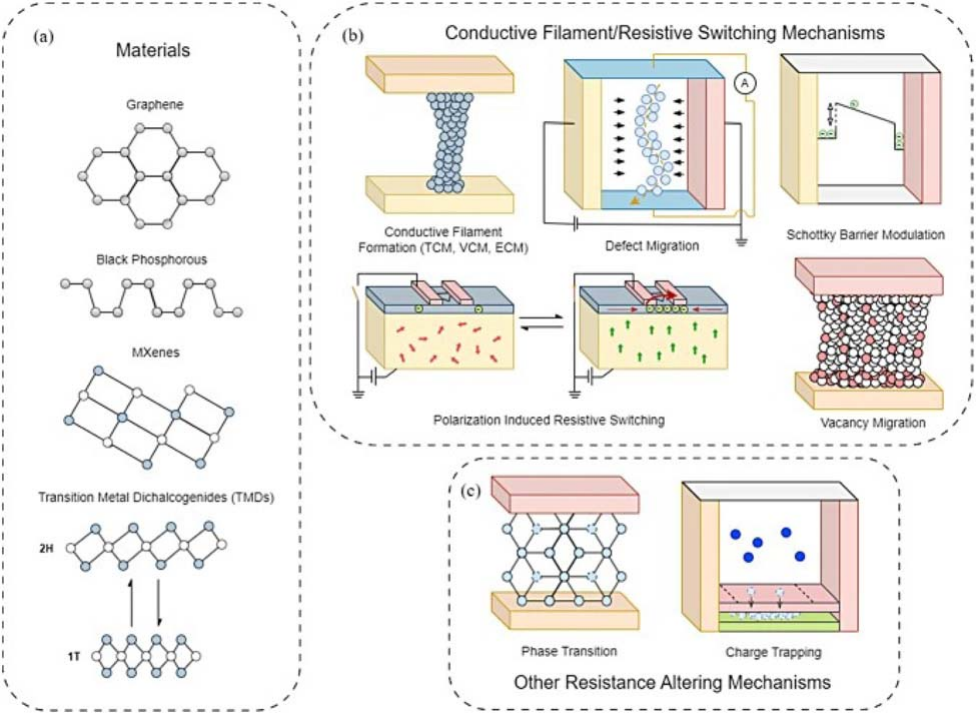
Two-dimensional material-based memristive devices. (a) Representative 2D materials including graphene, black phosphorus, MXenes, and transition metal dichalcogenides (TMDs). (b) Major resistance-altering mechanisms such as conductive filament formation (TCM, VCM, ECM), defect migration, Schottky barrier modulation, polarization-induced switching, and vacancy migration. (c) Other resistance modulation mechanisms including phase transition and charge trapping that do not necessarily rely on conductive filament formation, reproduced from ref. 13, J. Panisilvam *et al.*, *Materials Today Electronics*, 2023, 3, 100 017, licensed under CC BY 4.0, copyright © 2024.

Fig. 7(a) presents representative 2D materials investigated for memristive applications, including graphene, black phosphorus, MXenes, and transition metal dichalcogenides (TMDs). Their layered crystal structures allow precise thickness control down to the atomic scale and provide strong surface sensitivity. Graphene is widely used because of its excellent carrier mobility and mechanical stability, while TMDs such as MoS_2_ and MoTe_2_ offer tunable bandgaps and controllable interface properties suitable for resistive switching devices. The switching behavior of 2D-material memristors depends strongly on interface chemistry and defect dynamics. Fig. 7(b) summarizes several resistance-modulating mechanisms reported in these systems, including thermochemical (TCM), valence change (VCM), and electrochemical metallization (ECM) processes associated with conductive filament formation. In addition, defect migration, vacancy transport, Schottky barrier modulation, and polarization-induced switching contribute to resistance modulation depending on material composition and electrode configuration. Unlike many conventional oxide memristors that primarily depend on filament growth and rupture, 2D materials frequently exhibit mixed or interface-controlled switching behavior. The atomically sharp interfaces and reduced dimensionality of these materials allow stronger control over carrier injection and local electric-field distribution. Consequently, resistance modulation may occur through interfacial charge transfer or barrier-height variation rather than exclusively through stochastic conductive filament formation.

Fig. 7(c) further illustrates non-filamentary mechanisms such as phase transition and charge trapping. These switching modes are particularly attractive because they often produce smoother resistance modulation, reduced device-to-device variability, and improved switching uniformity compared with random filamentary conduction.^13^ Charge-trapping mechanisms may arise from localized interface states or defects, whereas phase-transition behavior results from reversible changes in crystal structure and electronic configuration. The unique electronic structure and interface tunability of 2D materials therefore provide opportunities for engineering memristive devices with lower power consumption, multi-level resistance states, and enhanced scalability. Because of these advantages, 2D-material memristors are increasingly considered promising candidates for neuromorphic computing, flexible electronics, and future CMOS-compatible memory and computing platforms.^11–13^

### 3.2 Metal oxide materials

Metal oxides remain the most extensively investigated material systems for memristive devices because of their simple fabrication, excellent thermal stability, and strong compatibility with CMOS technology. Common oxide materials include HfO_2_, TiO_2_, Ta_2_O_5_, ZnO, Al_2_O_3_, and NiO, each exhibiting distinct defect chemistry and switching characteristics. Among these, HfO_2_ and TiO_2_ have received considerable attention owing to their scalability, stable switching behavior, and suitability for high-density memory integration.^11,12^

In oxide-based memristors, resistive switching is primarily governed by oxygen-vacancy migration and conductive filament formation. Under an applied electric field, oxygen ions move away from their lattice positions, generating oxygen vacancies that act as electrically conductive defect sites. The migration and accumulation of these vacancies eventually form conductive filaments, switching the device from a high-resistance state (HRS) to a low-resistance state (LRS). Reversal of the applied polarity or local oxidation can rupture these conductive pathways and restore the HRS. Consequently, switching behavior strongly depends on defect concentration, filament stability, and electrode–oxide interface properties.

Different oxide materials exhibit distinct switching characteristics. HfO_2_-based devices are widely reported for their excellent endurance, low operating voltage, and compatibility with advanced CMOS fabrication. TiO_2_ memristors gained significant attention following the experimental demonstration of memristive behavior by Strukov *et al.*, establishing TiO_2_ as a model system for filamentary switching studies.^2^ Similarly, Ta_2_O_5_ devices often demonstrate improved switching uniformity and reduced variability because of better control over oxygen-vacancy distribution.

**Fig. 8:**
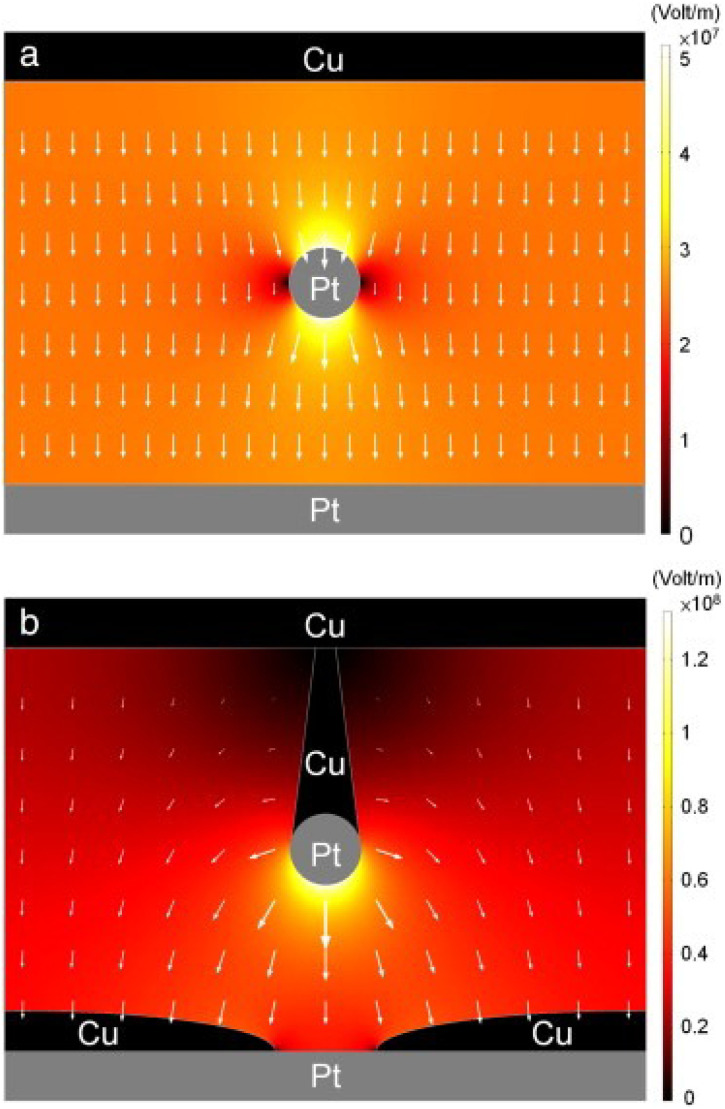
Simulation of electric field distribution in a Cu/Pt-nanoparticle-embedded SiO_2_/Pt structure. (a) Electric field distribution in the initial resistance state (IRS). (b) Electric field concentration after Cu filament formation above the Pt nanoparticle, reproduced from ref. 23 with permission from Elsevier, C.-Y. Liu, J.-J. Huang and C.-H. Lai, “Resistive switching characteristics of a Pt nanoparticle-embedded SiO_2_-based memory”, *Thin Solid Films*, 2013, **529**, 107–110, copyright 2013.

Fig. 8(a) illustrates the electric-field distribution in the initial resistance state (IRS) of the Cu/Pt-nanoparticle-embedded SiO_2_/Pt device. The electric field is relatively uniform throughout the oxide layer but becomes locally enhanced around the Pt nanoparticle. This localized enhancement is particularly important because stronger electric fields facilitate ion migration and accelerate defect generation.^23^

Following conductive filament formation, the electric-field profile changes significantly, as shown in Fig. 8(b). The field becomes concentrated along the Cu filament formed above the Pt nanoparticle, establishing a preferential current-conduction path and stabilizing the low-resistance state. These simulations highlight the strong coupling between local field enhancement, defect dynamics, and filament evolution during switching.^23^

To investigate switching mechanisms and material reliability, structural and morphological characterization techniques play an essential role. X-ray diffraction (XRD) identifies crystal phases and structural ordering, scanning electron microscopy (SEM) examines surface morphology, transmission electron microscopy (TEM) provides nanoscale cross-sectional imaging, atomic force microscopy (AFM) evaluates surface roughness, and Raman spectroscopy detects structural modifications and local bonding changes. These characterization methods are essential for correlating oxide microstructure and defect distribution with electrical performance. However, as device dimensions continue to shrink, maintaining precise control over defect concentration and filament reproducibility remains a major challenge for scalable oxide-based memristive technologies.^9,11,12^

### 3.3 Bulk materials

Bulk and hybrid material systems represent an important category of memristive materials in which resistive switching is governed by structural transformation, polarization reversal, or defect-assisted transport within relatively thicker active layers. Unlike atomically thin 2D materials, bulk systems generally exhibit stronger volume-driven switching behavior and offer greater flexibility in engineering multilayer structures and heterointerfaces. Common examples include phase-change materials such as Ge_2_Sb_2_Te_5_ (GST) and GeTe, as well as ferroelectric oxides including HfZrO_2_ and PbZrTiO_3_.^32,33^

**Fig. 9:**
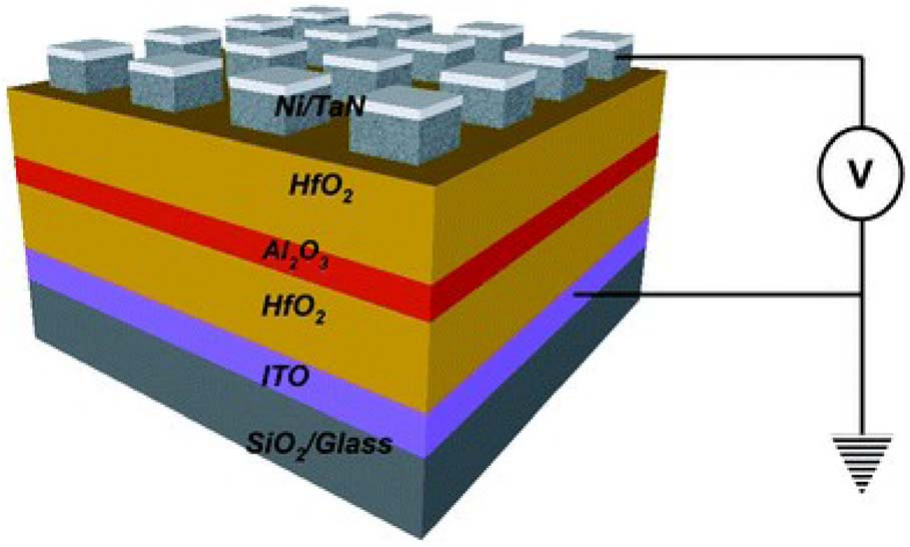
Schematic of Ni/TaN/HfO_2_/Al_2_O_3_/HfO_2_/ITO RRAM device structure with TaN top and ITO bottom electrodes, reproduced from ref. 33, E. A. Khera, C. Mahata, M. Imran, N. A. Niaz, F. Hussain, R. M. A. Khalil, U. Rasheed and S. Kim, “Improved resistive switching characteristics of a multi-stacked HfO_2_/Al_2_O_3_/HfO_2_ RRAM structure for neuromorphic and synaptic applications: experimental and computational study”, *RSC Advances*, 2022, **12**, 11649–11656, licensed under CC BY-NC 3.0 Unported Licence, copyright © 2022.

Fig. 9 illustrates a multilayer memristive device consisting of Ni/TaN/HfO_2_/Al_2_O_3_/HfO_2_/ITO layers fabricated on a SiO_2_/glass substrate. In this structure, Ni/TaN acts as the top electrode, ITO serves as the bottom electrode, and the stacked oxide layers form the active switching region. The introduction of multiple dielectric layers is particularly important because it allows improved electric-field distribution, defect confinement, and enhanced control of filament formation compared with single-layer oxide systems.^33^

Bulk material switching mechanisms vary depending on material chemistry and structural properties. In phase-change materials such as GST and GeTe, resistance modulation occurs through reversible transitions between amorphous and crystalline phases. These structural transformations produce significant conductivity differences and enable stable multilevel memory states. However, thermal programming and heat dissipation often become critical considerations in such devices.

Ferroelectric bulk materials operate through a different mechanism involving polarization reversal. Materials such as HfZrO_2_ and PbZrTiO_3_ alter internal electric fields through domain switching, thereby modulating carrier transport and resistance states. Compared with filamentary oxide devices, ferroelectric systems generally exhibit improved switching uniformity and reduced stochastic variability because resistance modulation is governed by polarization dynamics rather than random conductive-path formation.

The multilayer HfO_2_/Al_2_O_3_/HfO_2_ device shown in Fig. 9 demonstrates how hybrid bulk structures can combine the advantages of oxide switching with improved defect engineering.^33^ The Al_2_O_3_ interlayer functions as a barrier and field-modulation layer, helping regulate defect migration and stabilize switching behavior. Such multilayer engineering is increasingly explored to improve endurance, retention, and resistance-state stability in advanced memristive systems.

Structural and phase characterization plays a critical role in understanding bulk-material switching. X-ray diffraction (XRD) identifies crystalline phases and structural transitions, Raman spectroscopy detects bonding and phase evolution, scanning electron microscopy (SEM) evaluates morphology, transmission electron microscopy (TEM) investigates grain boundaries and interfaces, and atomic force microscopy (AFM) measures surface roughness. Since switching strongly depends on crystallinity, interface quality, and defect distribution, maintaining precise structural control remains essential for reliable bulk memristor performance.

A comparative summary of the major material classes discussed in this section is presented in Table 2.

**Table 2:** Comparative analysis of major memristor material systems and their switching characteristics

Material type	Representative materials	Switching mechanism	Advantages	Challenges
2D materials	MoS_2_, graphene, MXenes^13,34^	Schottky barrier modulation, charge trapping, defect migration^13^	Atomic thickness, tunable electronic properties, low-power operation	Defect control, interface sensitivity, fabrication complexity^30^
Metal oxides	HfO_2_, TiO_2_, Ta_2_O_5_, ZnO^2,11^	Oxygen-vacancy migration and conductive filament formation^3,12^	CMOS compatibility, stable switching, high scalability	Cycle variability and filament instability^28^
Bulk/Hybrid materials	GST, GeTe, HfZrO_2_, PbZrTiO_3_ (ref. 32 and 33)	Phase transition, polarization switching, hybrid transport^25,27^	High endurance, multilevel switching, structural stability	Thermal management and fabrication complexity^32^

## 4 Fabrication of memristor structures

The fabrication process plays a crucial role in determining the electrical behavior, reliability, and scalability of memristive devices. Beyond material selection and device architecture, fabrication conditions strongly influence defect generation, interface quality, film uniformity, and conductive filament formation. Consequently, precise control over fabrication parameters is essential for achieving reproducible switching characteristics and long-term device stability.

Memristor devices are generally fabricated using established semiconductor microfabrication techniques such as photolithography, sputtering, chemical vapor deposition (CVD), electron-beam evaporation, and atomic layer deposition (ALD). These methods provide precise control over switching-layer thickness, electrode geometry, surface morphology, and device dimensions. Since resistive switching mechanisms are highly sensitive to nanoscale structural variations, fabrication precision becomes particularly important for reliable device operation.

Different classes of memristors employ distinct fabrication strategies depending on their material system and switching mechanism. Oxide-based memristors commonly utilize sputtering or ALD to deposit uniform dielectric layers, whereas conductive-bridge memristors (CBRAM) often require controlled metal-ion diffusion and electrode engineering. Similarly, valence-change memristors (VCM) depend strongly on oxygen-vacancy distribution and oxide stoichiometry, while two-dimensional material memristors involve transfer or layer-growth techniques to preserve atomically thin interfaces.^3,5^

Fabrication methods therefore influence memristor performance not only through dimensional control but also by regulating defect chemistry and interfacial properties. Parameters such as deposition temperature, chamber pressure, precursor chemistry, and post-deposition annealing significantly affect switching voltage, endurance, retention, and variability. As memristor dimensions continue to scale toward nanoscale integration, maintaining uniform fabrication and minimizing process-induced defects remain critical challenges for CMOS-compatible and high-density memory technologies.

In addition to film thickness control and dimensional accuracy, practical memristor fabrication must address challenges associated with electrode diffusion, interface contamination, wafer-scale variability, and process repeatability. Uncontrolled metal diffusion can alter conductive filament formation and switching stability, while interface contamination may degrade carrier transport and increase device-to-device variability. Furthermore, maintaining uniform material properties and reproducible switching behavior across large wafer areas remains a significant manufacturing challenge. Therefore, precise process control, defect management, and repeatable fabrication methodologies are essential for achieving reliable large-scale VLSI integration of memristive devices.^18,35^

This section discusses the major fabrication techniques employed for memristive devices and highlights how process conditions influence structural quality and electrical performance.

### 4.1 Lithography-based memristor fabrication

Lithography is one of the most important fabrication techniques employed in memristor technology because it defines device geometry, electrode dimensions, and alignment accuracy. Precise patterning is essential for nanoscale memristors, particularly in crossbar architectures where switching behavior and device density strongly depend on feature size and electrode overlap. Consequently, lithography directly influences scalability, electrical uniformity, and integration capability in memristive systems.

**Fig. 10:**
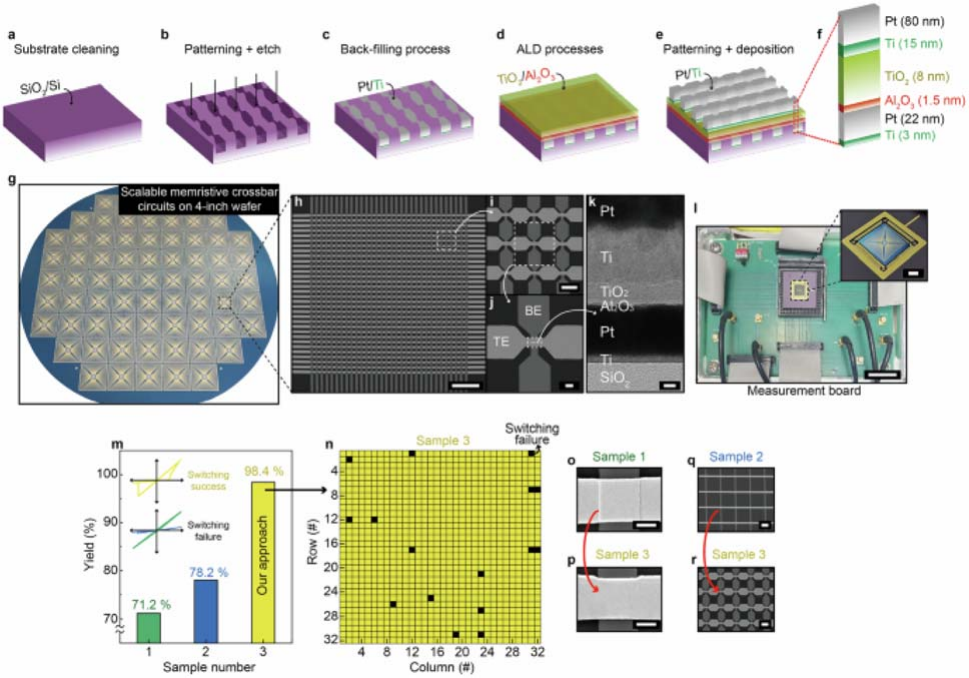
Photolithography process used in memristor fabrication showing photoresist coating, UV exposure, development, and electrode patterning. (a)–(e) Schematic of different fabrication steps, (f) zoomed schematic of the memristor, (g) top view of the optical image, (h)–(j) top views of the SEM images, (k) cross-sectional high resolution TEM image of the junction, (l) image of a measurement board, (m) histogram of the device yield as function of sample number, (n) yellow shows successful device and black shows a failure, (o) and (p) SEM magnified image at a cross-point, (q) and (r) top view of SEM image of a sub array, reproduced from ref. 36, H. Kim *et al.*, “Wafer-scale fabrication of memristive passive crossbar circuits for brain-scale neuromorphic computing”, *Nature Communications*, 2025, **16**, Article 7083, licensed under CC BY 4.0, copyright © 2025.

The photolithography sequence commonly used in memristor fabrication is illustrated in Fig. 10. Fabrication generally begins with substrate preparation, where silicon wafers coated with thermally grown SiO_2_ are widely employed owing to their excellent insulation and compatibility with semiconductor processing. Surface cleaning using acetone, isopropyl alcohol, and deionized water is performed to remove organic contamination and improve film adhesion.^36^

Following substrate preparation, a positive photoresist is spin-coated to obtain a uniform resist layer. Spin speed and resist viscosity determine film thickness and ultimately affect pattern resolution. The coated wafer is subsequently soft-baked, typically between 90 °C and 120 °C, to evaporate residual solvent and stabilize the resist layer before exposure.

As shown in Fig. 10, ultraviolet (UV) exposure through a patterned photomask transfers the desired electrode geometry onto the photoresist. During development, exposed resist regions are selectively removed, producing well-defined nanoscale patterns. Bottom electrodes such as Pt, Ti, or Au are then deposited using sputtering or evaporation, followed by a lift-off process that removes excess metal and preserves the patterned structure.^36^

In advanced nanoscale fabrication, pattern-transfer and etching processes must be carefully controlled because they can introduce surface damage, interface degradation, and additional defect states within the active device region 36. Such process-induced damage may alter conductive filament formation, increase switching variability, and reduce long-term device reliability. Consequently, minimizing etching-induced defects and preserving interface integrity are important considerations for achieving reproducible memristor performance and CMOS-compatible large-scale integration.^5,28^

After electrode formation, the active switching layer—commonly TiO_2_, HfO_2_, or related oxides—is deposited using sputtering or atomic layer deposition (ALD). Finally, the top electrode is patterned and deposited to complete the memristor stack. The accuracy of lithographic alignment during these stages strongly influences switching-area definition, current distribution, and filament localization.

The importance of lithography in memristor fabrication is demonstrated in the Pt/TiO_2_/Pt device reported by Strukov *et al.*, where photolithography and sputtering were used to fabricate one of the earliest experimentally verified memristors.^2^ In such oxide devices, switching behavior is associated with oxygen-vacancy migration and conductive filament formation. Similarly, high-density crossbar memristors such as Pt/HfO_2_/TiN structures have been fabricated using lithographic patterning and ALD to achieve improved scalability and dense memory integration.^5^

Although photolithography provides excellent reproducibility and compatibility with CMOS manufacturing, scaling below deep-submicrometer dimensions introduces challenges such as edge roughness, alignment error, and process variability. Therefore, continued advances in lithographic resolution and pattern-transfer precision remain important for realizing reliable and large-scale memristive architectures.

### 4.2 Chemical vapor deposition (CVD)-based memristor fabrication

Chemical vapor deposition (CVD) is widely employed for the synthesis of high-quality thin films and two-dimensional materials because it enables excellent control over film thickness, crystallinity, and large-area uniformity.

Among various fabrication techniques, CVD is particularly important for producing graphene- and MoS_2_-based memristive devices where interface quality and defect concentration strongly influence switching behavior.

In the CVD process, precursor gases are introduced into a high-temperature reaction chamber where chemical reactions occur on the substrate surface, leading to the formation of atomically thin or polycrystalline films. Growth conditions such as deposition temperature, precursor concentration, chamber pressure, and carrier-gas flow rate strongly affect grain size, layer thickness, and defect density. Consequently, electrical characteristics of the resulting memristor depend not only on material composition but also on precise process optimization.

**Fig. 11:**
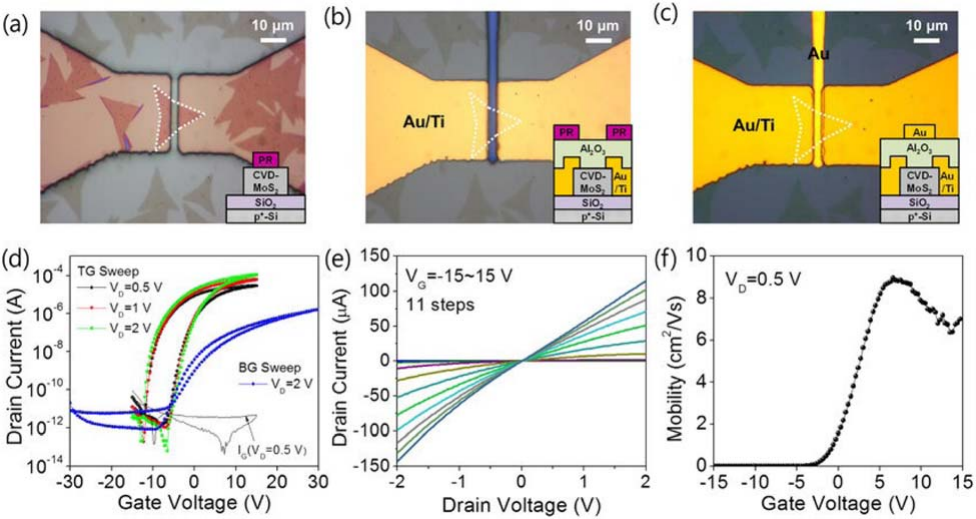
CVD-grown MoS_2_ device fabrication and electrical characterization. (a) Optical image after CVD growth and pattern definition. (b) Au/Ti contact deposition on MoS_2_ channel. (c) Completed Au/Ti electrode structure. (d) Transfer characteristics under different drain bias. (e) Output characteristics at various gate voltages. (f) Field-effect mobility variation with gate voltage, reproduced from ref. 22 with permission from IOP Publishing, H. Kwon, P. J. Jeon, J. S. Kim, T.-Y. Kim, H. Yun, S. W. Lee, T. Lee and S. Im, “Large scale MoS_2_ nanosheet logic circuits integrated by photolithography on glass”, *2D Materials*, 2016, **3**, 044001, copyright © 2016 IOP Publishing Ltd.

The fabrication and electrical characterization of a CVD-grown MoS_2_ device are illustrated in Fig. 11. Fig. 11(a) shows the optical image obtained after CVD growth and device pattern definition. Fig. 11(b) and (c) illustrate Au/Ti contact deposition on the MoS_2_ channel and the completed device structure. These fabrication stages are critical because metal–semiconductor contact quality significantly affects carrier injection and interface-controlled switching behavior.^22^

The electrical characteristics of the fabricated device are presented in Fig. 11(d)–(f). The transfer characteristics shown in Fig. 11(d) demonstrate strong gate-dependent modulation of drain current, confirming effective electrostatic control of carrier transport. Fig. 11(e) presents output characteristics exhibiting both linear and saturation regions typical of field-effect transport, while the field-effect mobility variation shown in Fig. 11(f) indicates efficient charge transport within the CVD-grown MoS_2_ layer.^22^

For memristive applications, CVD-grown 2D materials provide several advantages compared with conventionally sputtered oxide films. Atomically sharp interfaces and tunable electronic properties allow resistance modulation through charge trapping, defect migration, and Schottky barrier control rather than solely relying on stochastic filament formation. Such interface-controlled switching may improve switching uniformity and reduce variability.

Graphene oxide memristors with Au/GO/Au structures have demonstrated stable bipolar switching behavior associated with oxygen-containing functional groups and charge transport modulation.^8^ Similarly, MoS_2_ devices fabricated using CVD exhibit switching characteristics influenced by vacancy migration, interface states, and defect-assisted transport.^34^ However, precise control of intrinsic defects and grain boundaries remains a significant challenge because small structural variations can substantially alter switching voltage, retention, and long-term device reliability.

From a VLSI integration perspective, the compatibility of CVD-grown materials with conventional CMOS manufacturing must also be considered. Although CVD enables the synthesis of high-quality two-dimensional materials with excellent structural uniformity, many growth processes require elevated temperatures that may exceed standard back-end-of-line (BEOL) thermal budgets. Consequently, the development of low-temperature growth, transfer, and integration techniques remains important for achieving CMOS-compatible implementation of 2D-material-based memristive devices while preserving device performance and manufacturing reliability. In addition to thermal compatibility, wafer-scale uniformity and process repeatability remain important challenges for industrial implementation. Variations in grain size, defect density, and layer thickness across large-area substrates can lead to device-to-device variability and inconsistent switching behavior. Therefore, achieving reproducible wafer-scale growth and maintaining uniform material quality are essential requirements for large-scale manufacturing of CVD-based memristive devices.^22,34^

Therefore, although CVD offers excellent scalability and film-quality control, continued optimization of growth parameters and interface engineering remains essential for reliable and large-area fabrication of 2D-material memristive devices.

### 4.3 Sputtering-based memristor fabrication

Sputtering is one of the most widely employed thin-film deposition techniques for memristor fabrication because it offers excellent film uniformity, strong adhesion, and compatibility with CMOS processing. Both metallic electrodes and oxide switching layers can be deposited using sputtering, making it particularly suitable for large-area and high-density memristive devices.

Fabrication using sputtering generally begins with substrate cleaning to remove contamination and improve film adhesion. Bottom electrodes such as Pt, TiN, or TaN are commonly deposited using DC sputtering, whereas oxide switching layers including TiO_2_, HfO_2_, and Ta_2_O_5_ are frequently grown using RF sputtering. During sputtering, energetic ions generated in a plasma bombard the target material and eject atoms, which subsequently condense on the substrate surface to form thin films. Film thickness, morphology, and defect concentration are controlled by adjusting deposition power, chamber pressure, substrate temperature, and deposition duration.

After deposition of the switching layer, top electrodes such as Pt, Ag, or Cu are deposited to complete the memristor stack. Since resistive switching strongly depends on oxygen-vacancy concentration and interface quality, sputtering conditions significantly influence filament formation, switching voltage, and long-term reliability.

Oxide-based memristors fabricated using sputtering have demonstrated stable switching performance in several material systems. Pt/TiO_2_/Pt devices fabricated using sputtering exhibit resistive switching governed by oxygen-vacancy migration and conductive filament formation.^2^ Similarly, Ta/Ta_2_O_5_/Pt devices demonstrate reliable non-volatile switching behavior,^37^ while sputtered HfO_2_ memristors are widely reported for low-power operation and good endurance characteristics.^3^ Despite these advantages, maintaining uniform defect distribution and minimizing process-induced variability remain important fabrication challenges.

**Fig. 12:**
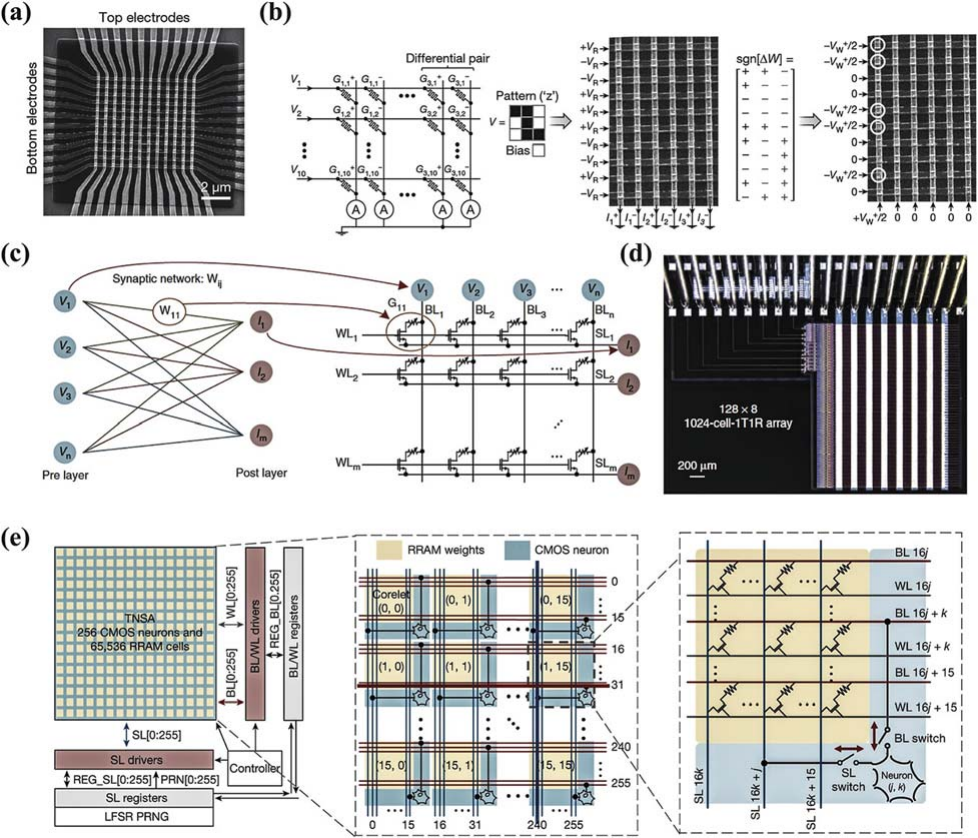
Compute-in-memory architecture based on RRAM crossbar arrays. (a) Optical micrograph of memristor crossbar array showing top and bottom electrodes. (b) Differential pair sensing scheme and pattern mapping. (c) Synaptic network implementation using crossbar architecture. (d) Optical image of fabricated 128 × 8 (1024-cell) 1T1R array. (e) System-level integration of RRAM crossbar with CMOS neuron and control circuitry, reproduced from ref. 17, L. Cai, L. Yu, W. Yue, Y. Zhu, Z. Yang, Y. Li, Y. Tao and Y. Yang, “Integrated Memristor Network for Physiological Signal Processing”, *Adv. Electron. Mater.*, 2023, **9**, 2300021, licensed under CC BY 4.0, copyright © 2023.

The relevance of sputtering for large-scale memristive integration is illustrated in Fig. 12. Fig. 12(a) presents an optical micrograph of a fabricated RRAM crossbar array, where perpendicular top and bottom electrodes intersect to form memristive cells. Such structures are commonly realized using sputtered electrode and oxide layers because sputtering provides uniform coverage and precise thickness control.^17^

To improve sensing accuracy during read operations, differential pair schemes such as the one shown in Fig. 12(b) are employed. This approach enhances signal discrimination and improves pattern-mapping reliability. Fig. 12(c) illustrates how crossbar architectures perform vector–matrix multiplication through analogue current summation, enabling highly parallel neuromorphic computation.^17^

The fabricated 128 × 8 (1024-cell) 1T1R array shown in Fig. 12(d) demonstrates practical implementation of large-scale sputtered memristor arrays. Incorporation of one transistor with each resistive element suppresses sneak-path currents and improves write selectivity. Finally, Fig. 12(e) presents system-level integration in which RRAM crossbar arrays are combined with CMOS neurons and peripheral circuitry.^17^

Such compute-in-memory architectures significantly reduce data transfer between processor and memory, thereby lowering energy consumption and improving computational throughput.^17^ Therefore, sputtering not only serves as an effective fabrication technique for memristive thin films but also enables scalable implementation of high-density and neuromorphic computing hardware.

### 4.4 Electron beam irradiation for defect engineering in 2D memristors

**Fig. 13:**
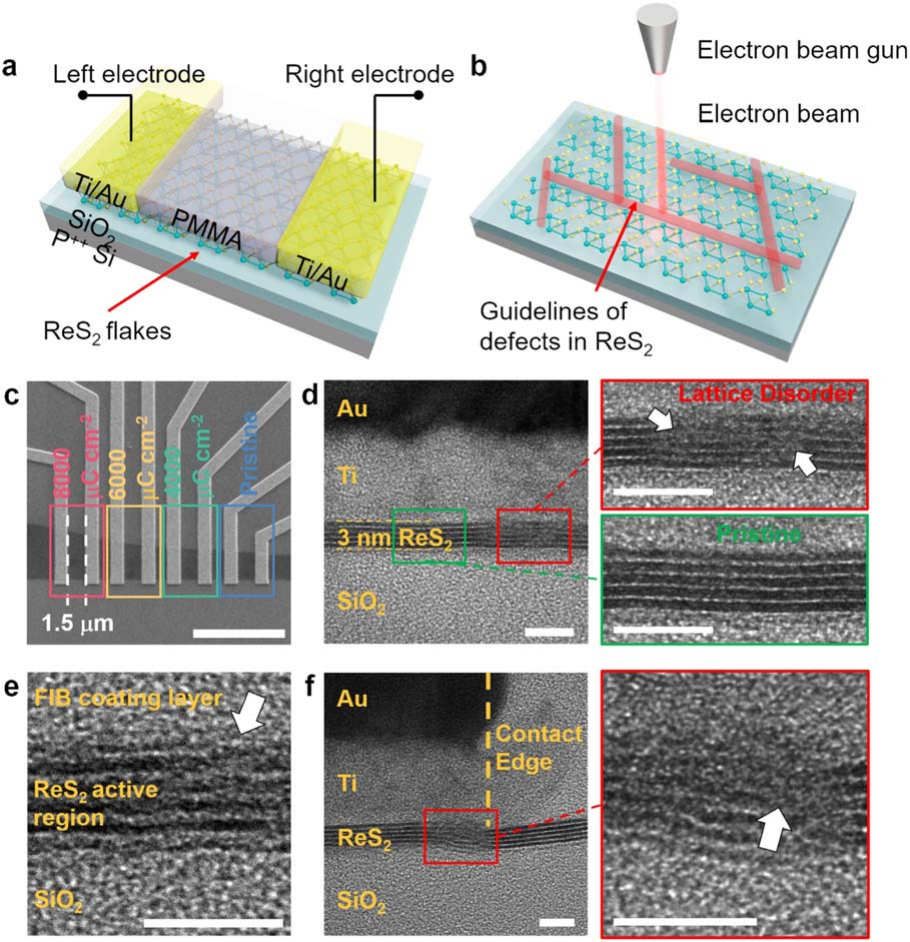
Electron-beam-irradiated ReS_2_ memristor devices. (a) Planar ReS_2_ memristor structure with Ti/Au electrodes. (b) Electron beam irradiation process for defect engineering. (c) SEM images of devices under different irradiation doses. (d) TEM cross-section showing lattice disorder and pristine regions. (e) High-resolution TEM image of ReS_2_ active region. (f) TEM image showing Ti/Au contact interface and defect region, reproduced from ref. 30, S. Li, B. Li, X. Feng, L. Chen, Y. Li, L. Huang, X. Fong and K.-W. Ang, “Electron-beam-irradiated rhenium disulfide memristors with low variability for neuromorphic computing”, *npj 2D Materials and Applications*, 2021, **5**, 1, licensed under CC BY 4.0, copyright © 2021.

In addition to conventional deposition techniques, electron-beam irradiation has emerged as an effective defect-engineering strategy for tailoring the electrical behavior of two-dimensional memristive materials. Unlike electron-beam evaporation, which is primarily employed for metal deposition, electron-beam irradiation intentionally modifies material structure by introducing controlled defects and localized lattice disorder. Such defect engineering is particularly important in atomically thin materials because switching characteristics strongly depend on vacancy concentration, interface states, and charge-trapping sites. Fig. 13(a) illustrates a planar ReS_2_ memristor fabricated with Ti/Au electrodes on a SiO_2_/Si substrate. As shown in Fig. 13(b), localized electron-beam irradiation is applied to selected regions of the ReS_2_ channel to intentionally generate structural defects. This approach enables precise spatial control of defect density without significantly altering the overall device geometry.

The influence of irradiation on device morphology and crystal structure is illustrated in Fig. 13(c)–(f). SEM images shown in Fig. 13(c) reveal structural changes occurring under different irradiation doses, indicating progressive modification of the active layer. The cross-sectional TEM image in Fig. 13(d) further confirms the coexistence of irradiated and pristine regions, where lattice disorder develops following electron-beam exposure. High-resolution TEM images presented in Fig. 13(e) and (f) reveal defect generation within the active ReS_2_ region and near the Ti/Au contact interface.^30^

These irradiation-induced defects strongly influence carrier transport and resistive switching behavior. Defect states act as localized trapping centers and modify charge transport pathways, enabling resistance modulation through defect-assisted conduction and interface-controlled transport mechanisms. Unlike conventional filamentary oxide memristors that rely primarily on conductive-path formation and rupture, irradiated ReS_2_ devices demonstrate switching behavior governed largely by defect distribution and interfacial modulation.

Controlled defect engineering therefore provides a powerful route for tuning memristor characteristics including switching voltage, analogue conductance modulation, and resistance-state stability. Such controllable switching behavior is particularly attractive for neuromorphic systems where gradual resistance tuning and analogue weight updates are required. Consequently, electron-beam irradiation offers not only a fabrication-support technique but also a functional method for engineering adaptive and highly tunable two-dimensional memristive devices.^30^

### 4.5 Atomic layer deposition (ALD)-Based memristor fabrication

Atomic layer deposition (ALD) is one of the most precise thin-film fabrication techniques employed for memristive devices because it enables atomic-scale control over film thickness and interface formation. Compared with conventional deposition methods, ALD provides excellent conformality, low surface roughness, and highly uniform ultrathin films, making it particularly suitable for nanoscale memristor fabrication and three-dimensional device architectures.

**Fig. 14:**
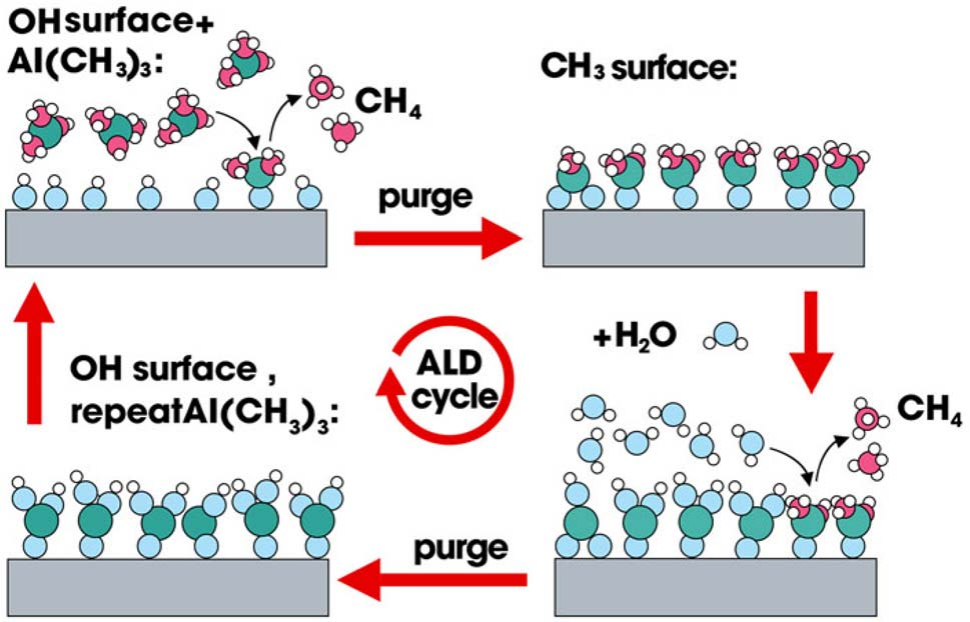
Atomic layer deposition process showing precursor pulse and purge cycle for deposition of switching layers such as HfO_2_ and Al_2_O_3_, reproduced from ref. 38, J. Guo, D. Wang, Y. Xu, X. Zhu, K. Wen, G. Miao, W. Cao, J. Si, M. Lu and H. Guo, “Secondary electron emission characteristics of Al_2_O_3_ coatings prepared by atomic layer deposition”, *AIP Advances*, 2019, **9**, 095303, copyright © 2019 AIP Publishing.

The ALD process, illustrated in Fig. 14, involves sequential precursor pulse and purge cycles. During deposition, precursor gases are introduced individually into the reaction chamber, where self-limiting surface reactions deposit a controlled atomic layer during each cycle. Subsequent purge steps remove excess precursor and reaction by-products before the next cycle begins. This layer-by-layer growth mechanism provides exceptional thickness precision and enables formation of defect-controlled dielectric films.^38^

In memristor fabrication, bottom electrodes such as TiN or Pt are deposited first, followed by deposition of the active switching layer using ALD. Materials including HfO_2_, Al_2_O_3_, and TiO_2_ are commonly fabricated using this technique because their switching characteristics strongly depend on nanoscale thickness and interface quality. After switching-layer formation, the top electrode is deposited to complete the device stack.

The advantages of ALD arise not only from dimensional control but also from its influence on defect chemistry and interfacial transport. Uniform film growth reduces thickness fluctuations and improves reproducibility of oxygen-vacancy distribution, which is critical for stable resistive switching. Consequently, ALD-fabricated memristors frequently demonstrate improved switching uniformity, lower leakage current, and enhanced endurance compared with devices fabricated using less controlled deposition approaches.

Recent fabrication strategies increasingly focus on simplifying processing while preserving device performance and scalability. Studies indicate that memristor arrays fabricated using optimized deposition sequences and simplified material systems can achieve improved uniformity and reduced manufacturing cost while maintaining reliable switching behavior.^14,18^ Such developments are particularly important for large-scale integration and industrial implementation.

As device dimensions continue to shrink, fabrication precision becomes increasingly critical. Nanoscale RRAM devices benefit from reduced operating power and faster switching because ultrathin switching layers and interfaces can be more precisely engineered.^14^ Nevertheless, despite its exceptional film-quality control, ALD still faces challenges related to slow deposition rate, precursor selection, and controlled defect engineering.

A comparative summary of the fabrication techniques discussed in this section is presented in Table 3.

**Table 3:** Comparative analysis of fabrication techniques used for memristor devices

Technique	Process type	Key advantages	Limitations	Typical memristor applications
Lithography	Patterning	High precision, nanoscale alignment, CMOS compatibility	Expensive and resolution-limited at advanced nodes	Electrode patterning and crossbar structures^5,8^
CVD	Thin-film growth	High-quality films, large-area uniformity, suitable for 2D materials	High temperature and process complexity	Graphene- and MoS_2_-based memristors^34,39^
Sputtering	Physical vapor deposition	Uniform oxide films, scalable deposition, strong adhesion	Defect distribution difficult to control	Oxide memristors and RRAM arrays^3,37^
ALD	Atomic-scale deposition	Excellent thickness control, conformal films, superior interfaces	Slow deposition rate and precursor limitations	Ultra-thin switching layers and nanoscale RRAM^40^
Electron-beam irradiation	Defect engineering	Localized defect control and tunable switching	High equipment cost and limited throughput	Research-scale 2D memristor devices^30^

## 5 Electrical characterization of memristive devices

Electrical characterization is essential for understanding the switching behavior, reliability, and operational mechanisms of memristive devices. Through electrical measurements, the transition between the high-resistance state (HRS) and low-resistance state (LRS) can be analyzed together with switching voltage, endurance, retention, and resistance stability. Since memristive devices employ diverse architectures and material systems, their electrical responses vary significantly depending on device structure, active material, and dominant switching mechanism.

Different memristor configurations, including Metal–Insulator–Metal (MIM), Metal–Insulator–Semiconductor (MIS), Metal–SiO_2_–Metal (MOM), planar devices, and crossbar architectures, exhibit distinct electrical characteristics because of variations in electric-field distribution, interface properties, and defect dynamics. In many oxide-based systems, switching behavior is primarily governed by conductive filament formation and rupture associated with oxygen-vacancy migration or electrochemical metallization.^3,10^ However, interface-controlled and non-filamentary switching mechanisms have also been reported, particularly in two-dimensional and ferroelectric memristive devices.

Apart from conventional filamentary conduction, certain nanoscale devices demonstrate atomic-scale switching behavior where conductance changes occur in discrete steps due to controlled atomic rearrangement or ion motion.^41^ Such quantized transport highlights the importance of nanoscale defect control and local electric-field engineering in determining memristor performance.

Experimental studies further demonstrate that electrical characteristics strongly depend on material composition and switching mechanism, directly influencing endurance, switching uniformity, and operational stability.^9^ Investigations involving TiO_2_-based memristors, oxide switching devices, and crossbar architectures reveal substantial differences in current–voltage (*I*–*V*) behavior, threshold voltage, and resistance modulation.^2,3,10,28,42^ These observations indicate that electrical performance cannot be attributed solely to device geometry or material selection; rather, it emerges from the combined influence of structure, defect dynamics, interface quality, and electric-field distribution.

Therefore, electrical characterization provides a direct means of correlating switching phenomena with underlying physical mechanisms and is essential for evaluating the suitability of memristive devices for memory, neuromorphic, and compute-in-memory applications.

### 5.1 Current–voltage (*I*–*V*) characterization with graph and explanation

#### 5.1.1 MIM memristor *I*–*V* characteristics

Current–voltage (*I*–*V*) characterization is one of the most important methods used to investigate the switching behavior of memristive devices. The electrical response provides information about resistance modulation, switching mechanism, and transport behavior inside the active layer. For Metal–Insulator–Metal (MIM) memristors, the *I*–*V* response typically exhibits a pinched hysteresis loop, which is considered a fundamental characteristic of memristive systems.

**Fig. 15:**
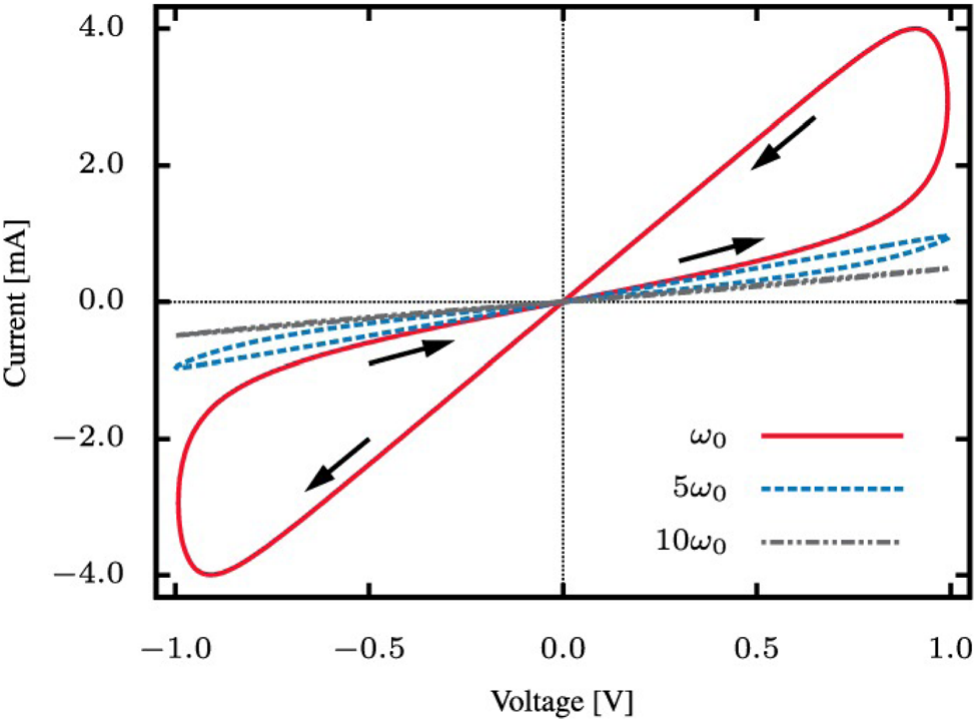
I–V hysteresis loop of TiO_2_ MIM memristor showing bipolar resistive switching behavior, reproduced from ref. 43, Tejinder Singh, “Hybrid Memristor-CMOS (MeMOS) based Logic Gates and Adder Circuits”, arXiv:1506.06735, copyright © 2015.

Fig. 15 shows the *I*–*V* hysteresis characteristics of a TiO_2_-based MIM memristor. The hysteresis loop passes through the origin, indicating memory-dependent resistance behavior. During voltage sweeping, the device switches between a high-resistance state (HRS) and a low-resistance state (LRS), demonstrating bipolar resistive switching.^43,44^

The observed switching behavior is mainly associated with oxygen-vacancy migration and conductive filament formation inside the TiO_2_ layer. Under an applied electric field, oxygen vacancies drift and accumulate, forming conductive pathways that reduce resistance and create the LRS. When the voltage polarity is reversed, these conductive paths are partially ruptured or redistributed, restoring the HRS. Therefore, resistance switching arises from reversible defect movement rather than permanent structural breakdown.

A notable feature of the memristor *I*–*V* response is its dependence on operating frequency. As shown in Fig. 15, the hysteresis loop is wider at lower frequency (*ω*_0_). Under these conditions, ionic species have sufficient time to respond to the applied electric field, enabling stronger defect migration and more pronounced filament evolution. Consequently, the device exhibits clear memory behavior and larger hysteresis area.^43,44^

As the excitation frequency increases (5*ω*_0_ and 10*ω*_0_), the hysteresis loop gradually decreases in size and approaches a nearly linear response. At higher frequencies, oxygen vacancies cannot fully follow the rapidly changing electric field, reducing filament growth and weakening resistance modulation. This frequency-dependent hysteresis shrinkage is widely recognized as a characteristic signature of memristive switching and confirms the important role of ionic transport in device operation.^2,43,45^

The *I*–*V* characteristics of TiO_2_ MIM memristors therefore demonstrate the close relationship between electrical response, defect dynamics, and filament evolution. Such electrical characterization is essential for understanding switching mechanisms and evaluating device suitability for non-volatile memory, neuromorphic computing, and compute-in-memory applications.

#### 5.1.2 MIS memristor switching characteristics

Metal–Insulator–Semiconductor (MIS) memristors exhibit diverse switching characteristics depending on the dominant transport mechanism and interface properties. Unlike conventional filament-dominated MIM devices, MIS structures often demonstrate both abrupt and gradual switching behavior because carrier transport is influenced by interfacial barrier modulation together with defect migration. These switching modes strongly affect device suitability for digital memory and neuromorphic applications.

**Fig. 16:**
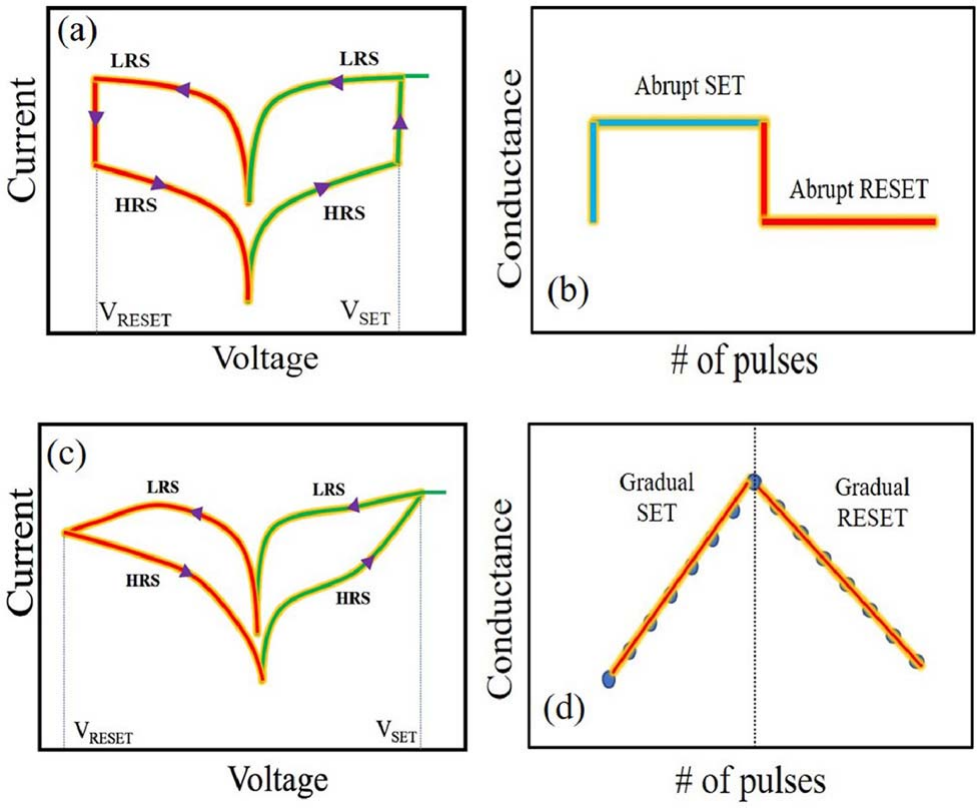
(a) Bipolar resistive switching showing abrupt SET and RESET transitions between HRS and LRS. (b) Conductance change with number of pulses showing abrupt switching. (c) Bipolar resistive switching showing gradual SET and RESET transitions. (d) Conductance change with number of pulses showing gradual switching behaviorm, reproduced from ref. 46, M. Asif and A. Kumar, “Resistive switching in emerging materials and their characteristics for neuromorphic computing”, *Materials Today Sustainability*, 2022, **18**, 100152, licensed under CC BY-NC-ND 4.0, copyright © 2022.

Fig. 16(a) presents bipolar resistive switching with abrupt SET and RESET transitions. Initially, the device remains in the high-resistance state (HRS). When the applied voltage reaches the SET threshold (*V*_SET_), the current rises sharply and the device switches to the low-resistance state (LRS). This sudden transition is associated with rapid conductive-path formation or strong interface-state modulation. During reverse bias, application of the RESET voltage (*V*_RESET_) causes a sudden reduction in current and restores the HRS, indicating rupture or destabilization of the conductive pathway.^46^

The conductance evolution corresponding to abrupt switching is shown in Fig. 16(b). Conductance increases rapidly during SET and decreases sharply during RESET, reflecting discrete resistance transitions. Such behavior is advantageous for binary memory operation because it provides clear distinction between HRS and LRS and enables reliable digital information storage.^46^

In contrast, Fig. 16(c) illustrates gradual bipolar switching behavior. Here, current changes progressively during both SET and RESET operations rather than undergoing abrupt transitions. This gradual resistance modulation is commonly attributed to controlled defect redistribution, partial filament evolution, or interface-mediated transport processes. Because switching occurs incrementally, multiple intermediate resistance states can be achieved.^46^

The pulse-dependent conductance response shown in Fig. 16(d) further demonstrates gradual switching behavior. Conductance increases stepwise during SET pulses and decreases gradually during RESET pulses, indicating analogue-like resistance modulation. Such continuous conductance tuning is particularly attractive for neuromorphic and synaptic computing systems because it enables analogue weight updates and learning behavior similar to biological synapses.^46^

The coexistence of abrupt and gradual switching therefore highlights the flexibility of MIS memristors. Abrupt switching is generally preferred for non-volatile digital memory owing to its strong ON/OFF distinction, whereas gradual switching provides improved programmability and multi-level conductance control required for artificial neural networks and adaptive computing systems.^2,45,46^

#### 5.1.3 MOM (SiO_2_-Based) memristor conduction mechanisms

**Fig. 17:**
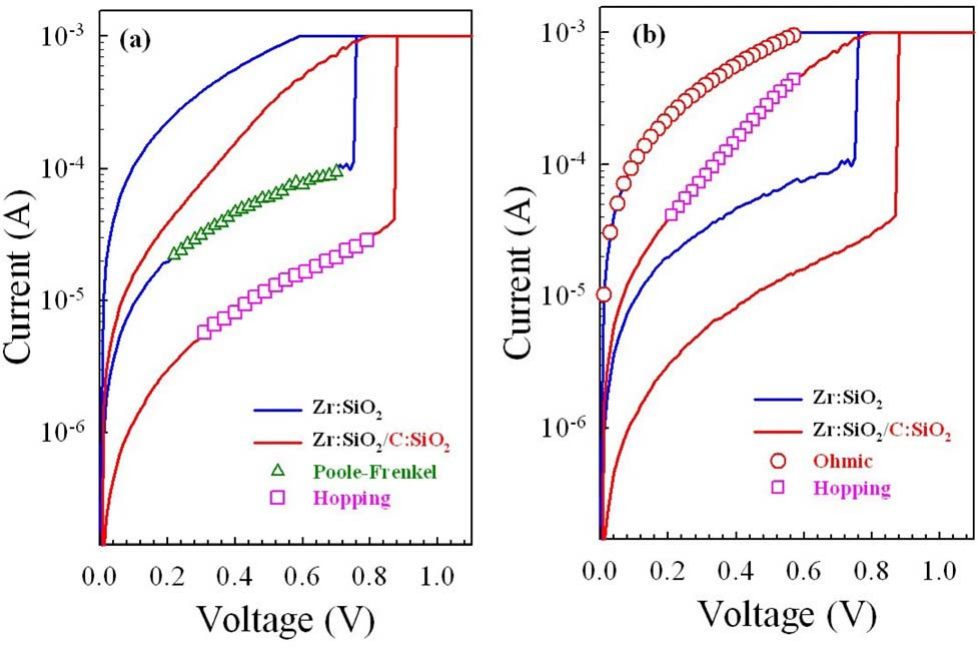
*I*–*V* characteristics of (a) Zr:SiO_2_ and (b) Zr:SiO_2_/C:SiO_2_ memristors showing ohmic, hopping, and Poole–Frenkel conduction mechanisms, reproduced from ref. 47, R. Zhang, K.-C. Chang, T.-C. Chang, T.-M. Tsai, K.-H. Chen, J.-C. Lou, J.-H. Chen, T.-F. Young, C.-C. Shih, Y.-L. Yang, Y.-C. Pan, T.-J. Chu, S.-Y. Huang, C.-H. Pan, Y.-T. Su, Y.-E. Syu and S. M. Sze, “High performance of graphene oxide-doped silicon oxide-based resistance random access memory”, *Nanoscale Research Letters*, 2013, **8**, 497, licensed under CC BY 2.0, copyright © 2013.

The electrical conduction behavior of SiO_2_-based memristors is governed by multiple transport mechanisms that become dominant under different electric-field conditions. Unlike ideal metallic conduction, current transport in dielectric switching layers strongly depends on trap states, defect distribution, and electric-field-assisted carrier transport. Fig. 17 presents the *I*–*V* characteristics of Zr:SiO_2_ and Zr:SiO_2_/C:SiO_2_ memristive devices, illustrating transitions between different conduction regimes.^47^

At low applied voltage, the device exhibits ohmic conduction where current increases linearly with voltage. In this regime, charge transport occurs primarily through thermally generated free carriers and uniformly distributed conductive paths. The linear *I*–*V* relationship indicates limited field enhancement and minimal influence of deep trap states.

As the applied voltage increases, hopping conduction becomes increasingly important. In this intermediate-field regime, charge carriers move between localized trap sites created by defects and impurities inside the SiO_2_ switching layer. Such hopping transport is strongly influenced by defect concentration and trap distribution, leading to nonlinear current response and enhanced field dependence.

At higher voltage, the dominant mechanism shifts toward Poole–Frenkel conduction, as observed in Fig. 17. Under strong electric fields, trapped electrons acquire sufficient energy to escape from localized trap states into the conduction band. This field-assisted trap ionization produces rapid current increase and pronounced nonlinear *I*–*V* characteristics.^45,47,48^ The Poole–Frenkel effect therefore reflects strong coupling between electric-field intensity and defect-assisted carrier transport.

The transition between ohmic, hopping, and Poole–Frenkel conduction mechanisms demonstrates the complex transport behavior of SiO_2_-based memristors. Such multi-regime conduction is particularly important because it directly influences switching voltage, leakage current, and resistance-state stability. Understanding these transport mechanisms is therefore essential for optimizing defect engineering and improving the electrical reliability of SiO_2_-based memristive devices.

#### 5.1.4 Planar memristor switching characteristics

Planar memristors differ from conventional vertical structures because current transport and resistive switching occur laterally along the active layer. This geometry provides improved access to the switching region and allows direct investigation of defect migration, filament evolution, and electric-field distribution. Consequently, planar devices are widely employed to study switching dynamics and nanoscale transport mechanisms.

**Fig. 18:**
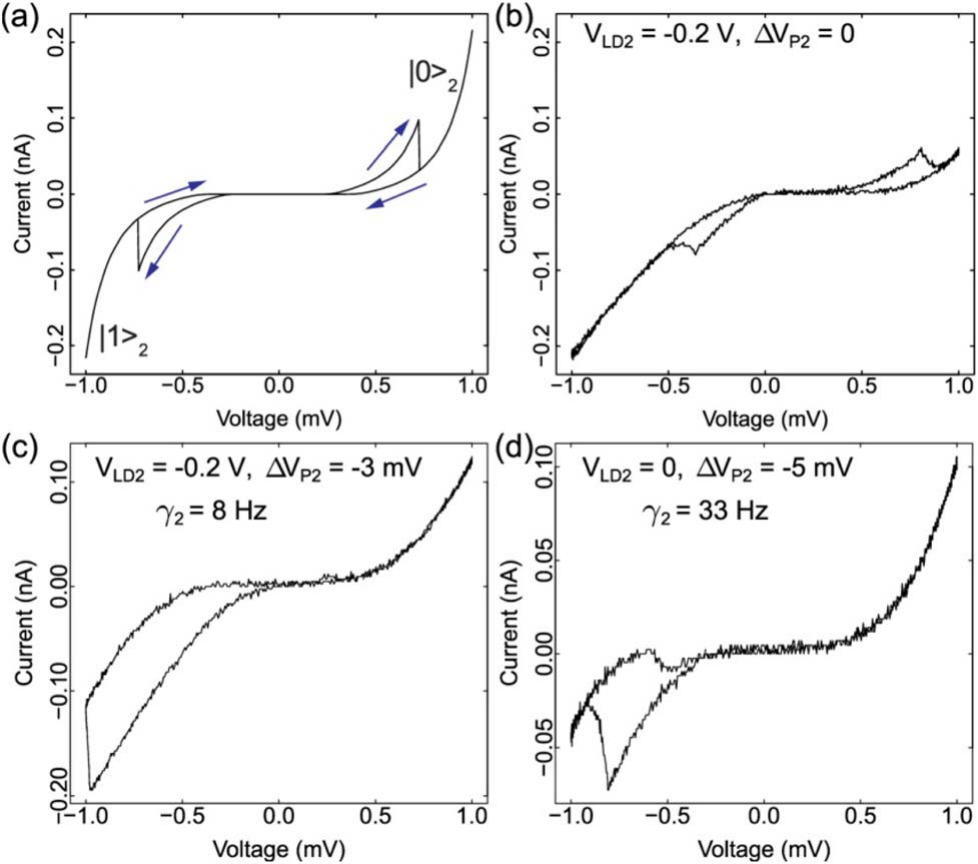
*I*–*V* characteristics of planar memristor showing frequency-dependent resistive switching behavior under different voltage conditions, (a) is a simulated curve, whereas (b)–(d) are measured *I*–*V* curves reproduced from ref. 49 with permission from the American Physical Society, Y. Li, G. W. Holloway, S. C. Benjamin, G. A. D. Briggs, J. Baugh and J. A. Mol, “Double quantum dot memristor”, *Physical Review B*, 2017, 96, 075446, copyright 2017.

Fig. 18 presents the electrical characteristics of a planar memristor under different operating conditions. Fig. 18(a) shows the characteristic pinched hysteresis loop passing through the origin, confirming memristive behavior. During voltage sweeping, the device switches between the low-resistance state (LRS, |0〉) and high-resistance state (HRS, |1〉), indicating non-volatile resistance modulation.^49^ Such switching behavior is commonly associated with electric-field-driven ion migration and conductive filament evolution inside the active switching layer.^1,2^

The electrical response under different bias conditions reveals strong dependence on both applied voltage and operating frequency. Fig. 18(b) presents the *I*–*V* characteristics measured at *V*_LD2_ = −0.2 V and Δ*V*_P2_ = 0, where current increases gradually and the hysteresis behavior remains relatively weak. This response corresponds to the initial resistance state in which conductive pathways are either absent or only partially developed, resulting in stable conduction without significant filament growth.^3^

A more pronounced hysteresis loop is observed in Fig. 18(c) for *V*_LD2_ = −0.2 V and Δ*V*_P2_ = −3 mV at operating frequency *γ*_2_ = 8 Hz. Under these conditions, ions and defects have sufficient time to respond to the electric field, promoting controlled filament formation and rupture. Consequently, resistance modulation becomes stronger and the memory effect is more clearly observed.^49^

Fig. 18(d) shows the electrical response at *V*_LD2_ = 0 and Δ*V*_P2_ = −5 mV with higher frequency *γ*_2_ = 33 Hz. The increased electrical excitation enhances ion transport and accelerates conductive-path evolution, leading to stronger nonlinear switching behavior and larger current response. This observation demonstrates the important role of electric-field strength and excitation conditions in controlling planar memristor performance.^49^

Overall, planar memristors exhibit nonlinear hysteresis and frequency-dependent switching behavior governed by defect migration and conductive-path evolution. Their lateral geometry, combined with stable resistive switching, makes them particularly useful for investigating nanoscale switching physics as well as for applications in non-volatile memory and neuromorphic computing systems.^45,49^

#### 5.1.5 Memristor crossbar array characteristics

Memristor crossbar arrays represent one of the most promising architectures for high-density non-volatile memory and neuromorphic computing systems. In this configuration, perpendicular top and bottom electrode lines intersect to form memristive cells at each cross-point, enabling extremely compact integration and large storage density. Because each cell functions as a programmable resistive element, crossbar arrays provide an efficient platform for both information storage and analogue computation.

**Fig. 19:**
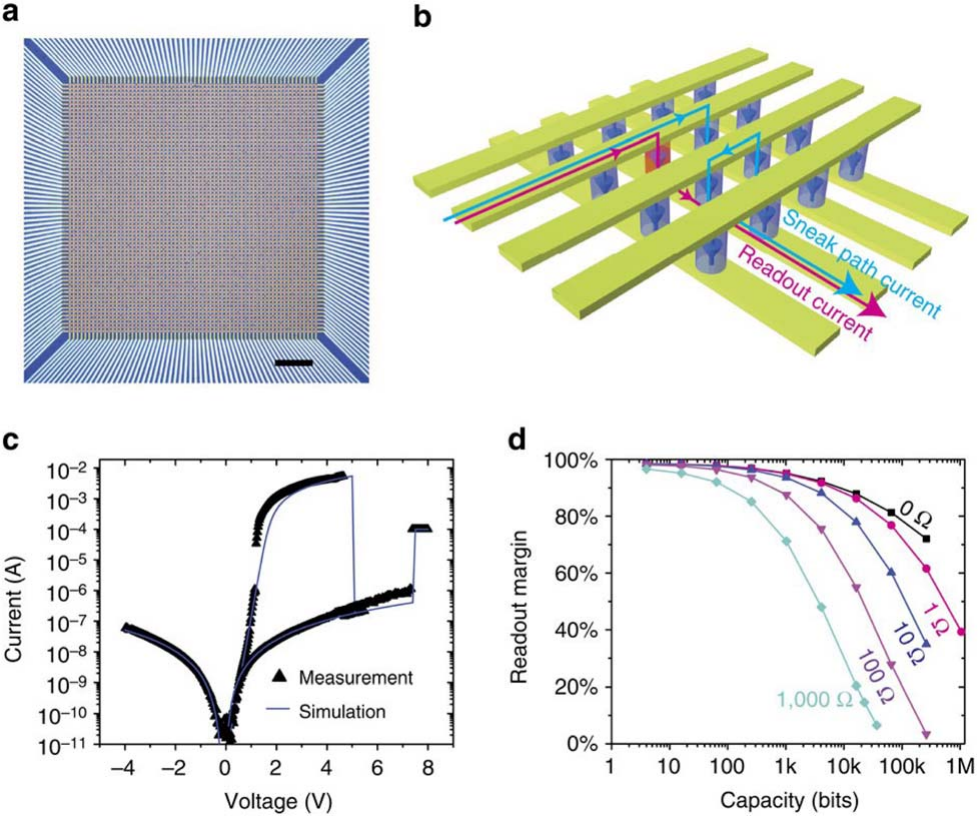
Memristor crossbar array showing (a) device structure, (b) sneak path current mechanism, (c) measured and simulated *I*–*V* characteristics, and (d) readout margin variation with array size, reproduced from ref. 50, C. Li, L. Han, H. Jiang, M.-H. Jang, P. Lin, Q. Wu, M. Barnell, J. J. Yang, H. L. Xin and Q. Xia, “Three-dimensional crossbar arrays of self-rectifying Si/SiO_2_/Si memristors”, *Nature Communications*, 2017, 8, 15 666, licensed under CC BY 4.0, copyright © 2017.

Fig. 19(a) illustrates the structure of a memristor crossbar array. Horizontal and vertical electrode lines form memory cells at their intersections, where each memristor stores information through programmable resistance states. The simple two-terminal geometry enables dense integration and makes crossbar structures highly attractive for large-scale memory systems and compute-in-memory architectures.^2,3,50^

Despite these advantages, crossbar arrays face an important challenge associated with sneak-path currents. As illustrated in Fig. 19(b), the desired read current flows through the selected cell, while parasitic currents simultaneously travel through neighboring unselected paths. These unwanted current paths distort the measured signal, reduce sensing accuracy, and become increasingly severe as array size grows.^45,50,51^ Consequently, sneak-path suppression remains one of the major limitations of passive crossbar systems.

The electrical characteristics shown in Fig. 19(c) provide further insight into device operation. The measured and simulated *I*–*V* responses exhibit nonlinear switching behavior associated with conductive filament formation and rupture. Good agreement between experimental and modeled characteristics validates the switching mechanism and confirms the suitability of the device model for predicting array-level behavior.^2,45,50^ Such nonlinear I–V characteristics are beneficial because they help suppress leakage currents and improve array selectivity.Array scalability is strongly influenced by readout margin, which determines the ability to distinguish between high-resistance and low-resistance states. Fig. 19(d) shows that readout margin decreases as array capacity increases due to the cumulative influence of sneak-path currents and parasitic resistance. However, larger HRS/LRS resistance ratios improve sensing reliability and enable more stable read operation in larger arrays.^3,45,50^

To further improve array reliability, one-transistor–one-resistor (1 T1R) and selector-assisted architectures are frequently employed to suppress leakage pathways and enhance read selectivity. Such approaches improve device-to-device uniformity and reduce read disturbance, particularly in highly scaled systems.

Crossbar memristors are particularly attractive for neuromorphic and compute-in-memory applications because they can directly perform analogue vector–matrix multiplication through Ohm's and Kirchhoff's laws. Instead of transferring data continuously between processor and memory, computation occurs inside the memory array itself, significantly reducing latency and energy consumption. Recent nanoscale crossbar systems have demonstrated improved switching uniformity, higher integration density, and enhanced computational efficiency compared with conventional memory architectures.^15,50^ Therefore, memristor crossbar arrays provide a scalable pathway toward next-generation memory and artificial intelligence hardware.

Table 4 presents a quantitative comparison of representative memristor technologies based on their electrical performance characteristics.

**Table 4:** Comparative analysis of representative memristor technologies based on electrical performance and reliability characteristics compiled from literature reports

Device type	Representative material/Stack	Operating voltage	ON/OFF ratio	Endurance	Retention	Switching behavior	Major reliability challenge
MIM oxide	TiO_2_, HfO_2_, Ta_2_O_5_ (ref. 2–4)	Moderate	High	Moderate–High	Good	Filamentary (VCM/ECM)	Filament instability and cycle variability^28,29^
2D material	MoS_2_, ReS_2_, graphene^13,30^	Low	Moderate	Moderate	Good	Interface/defect controlled	Defect and interface variability^30^
Phase-change	MoTe_2_, GST^25,32^	Moderate	High	High	Excellent	Structural phase transition	Thermal management and phase stability^25^
Ferroelectric	HfO_2_:Si, FeCAP-based devices^26,27^	Low	Moderate–High	Very high	Excellent	Polarization switching	Domain variability and material optimization^26^
Crossbar arrays	RRAM/1 T1R architectures^5,42^	Low–Moderate	Depends on device type	Array dependent	Good	Array-level resistive switching	Sneak-path current and read disturbance^50^

From a practical perspective, no single electrical parameter is sufficient to evaluate memristor performance. Reliable operation requires a balance between switching voltage, ON/OFF ratio, endurance, retention, power consumption, and device variability. As a result, different material systems and device architectures are often optimized for specific memory, neuromorphic, or compute-in-memory applications.

## 6 Process challenges and conclusion

Memristor technology has attracted considerable attention as a promising candidate for next-generation non-volatile memory, neuromorphic computing, and compute-in-memory systems because of its simple device structure, low operating power, and capability for analogue resistance modulation.^1,2,5^ Experimental demonstrations across oxide, two-dimensional, phase-change, and ferroelectric material systems have reported reliable resistive switching and high integration potential.^4,45^ However, despite these advantages, several fabrication and reliability challenges continue to limit large-scale commercial implementation.

One of the most critical challenges is device variability. In many oxide-based memristors, switching depends on the formation and rupture of nanoscale conductive filaments inside the switching layer.^3,8,29^ Small fluctuations in oxide thickness, defect density, interface quality, or fabrication conditions may significantly alter filament dynamics and switching behavior.^28,52^ Consequently, both device-to-device variability and cycle-to-cycle fluctuations are frequently observed in experimental studies.^10,45^ Although fabrication techniques such as sputtering, atomic layer deposition (ALD), and electron-beam processing provide improved control over film growth and interfaces, precise regulation of oxygen vacancies and nanoscale defects remains a major challenge.^16,32,40^

Reliability associated with endurance and retention presents another important limitation. Repeated switching cycles involving conductive-path formation and rupture gradually degrade the active layer and may introduce irreversible structural damage.^28^ Such degradation can result in increased switching variability, resistance drift, and eventual device failure after prolonged operation.^45,52^ Experimental evidence indicates that material stability, optimized interfaces, and carefully engineered device architectures are essential for improving long-term retention and cycling endurance.^32,37^ Therefore, defect engineering and interface optimization remain key research priorities for reliable memristive operation.

Filament instability further complicates practical implementation of memristive devices. In many metal–insulator–metal (MIM) systems, conductive pathways form stochastically and do not necessarily reappear at identical locations during repeated switching cycles.^4,29^ Such randomness produces unpredictable resistance states and limits switching reproducibility.^3,10^ Current research therefore focuses on improving filament localization through material selection, multilayer engineering, and controlled defect distribution.^12,32^

Large-scale crossbar arrays introduce additional system-level challenges, particularly sneak-path currents and read-disturb effects. Parasitic current leakage through neighboring cells reduces sensing accuracy and increases power consumption, thereby limiting array scalability.^42^ Selector devices, one-transistor–one-resistor (1 T1R) architectures, and advanced biasing techniques have been proposed to suppress leakage currents and improve array-level read margin.^5^ Nevertheless, balancing density, power efficiency, and read reliability remains a challenging design problem.

Compatibility with CMOS manufacturing is equally important for practical deployment.Memristor fabrication must satisfy back-end-of-line (BEOL) thermal budgets and integrate with standard semiconductor processing without degrading surrounding circuitry.^4,40^ High-temperature processing, contamination issues, and material incompatibilities may restrict large-scale integration. Consequently, low-temperature deposition techniques and CMOS-compatible material systems are increasingly emphasized for industrial implementation.

In addition to CMOS compatibility, practical memristor fabrication must address challenges associated with electrode diffusion, interface contamination, and wafer-scale process variability. Electrode diffusion can alter conductive filament formation and switching stability, while interface contamination may degrade carrier transport and increase device-to-device variability. Furthermore, maintaining uniform material properties and reproducible switching behavior across large wafer areas remains a significant manufacturing challenge. Therefore, precise process control, defect management, and repeatable fabrication methodologies are essential for achieving reliable large-scale VLSI integration of memristive devices.

In conclusion, memristor technology offers substantial opportunities for future memory and computing systems owing to its scalability, analogue programmability, and potential for neuromorphic and compute-in-memory architectures. This review has discussed major memristor structures, material systems, fabrication methods, and electrical characterization techniques together with their underlying switching mechanisms and performance characteristics. Comparative analysis indicates that no single device architecture presently satisfies all performance requirements simultaneously; instead, each material system exhibits unique advantages and limitations. Future progress therefore depends on improved defect engineering, interface control, reliable fabrication processes, and scalable array integration. Continued advances in these areas are expected to accelerate the transition of memristive devices from laboratory demonstrations toward practical VLSI and artificial-intelligence hardware applications.

### 6.1 Conclusion

The emergence of memristors as a highly viable platform in future applications of non-volatile memories and computing technologies is mainly due to the simplicity of two-terminal devices, their scalable size down to nano-scales, and the possibility of tuning resistance in an analog manner. In this review, different types of memristor structures such as MIM, MIS, SiO_2_, phase change, ferroelectric, planar, and crossbar have been discussed, along with the mechanism responsible for switching in each device type. It has been found that switching in a resistive manner can be achieved depending on several factors.

Comparison of various material systems that include metal oxides, 2D materials, and bulk/hybrid systems clearly shows that each has its own merits and demerits. Devices based on metal oxides are known for their CMOS compatibility and advanced fabrication methods; however, 2D materials have an upper hand in terms of better electrostatic control and adjustable interfaces. Likewise, phase change and ferroelectric memristors are favorable because of their ability to perform multi-level switching, higher endurance capacity, and reliable switching performance. Various fabrications techniques that include lithography, sputtering, CVD, electron beam technology, and ALD have also been discussed in detail.

Electrical characterizations described in this article show that the operation of memristors cannot be described only by their qualitative switching behavior. Several parameters including forming voltage, SET/RESET voltage, ON/OFF ratio, endurance, retention, switching energy, and variability of arrays contribute to the overall performance of memristive devices. Analysis shows that there is no memristive technology that satisfies all criteria for device performance at once, so device choice depends on the application.

Of all the material systems mentioned, ferroelectric memristors using HfO_2_ and HfO_2_/TiN using oxides are particularly appealing for incorporation into CMOS fabrication owing to their scalability, low-power characteristics, and compatibility with current fabrication technology. Additionally, their suitability for integration into BEOL makes them very suitable for future use as memory elements in neuromorphic circuits.

As seen from recent findings published in the literature, the improvement of switching uniformity and reliability is possible through proper material design and fabrication at nanoscale dimensions. The crossbar architecture and computing-in-memory systems have been instrumental in showcasing the power of memristors in the development of neuromorphic and artificial intelligence devices.^53–55^

Even as significant improvements have been made in these areas, however, there still exist critical issues relating to variability, endurance decay, filament behavior, sneak-paths, and large-scale integration in CMOS circuits. In order to resolve these problems, further work must be done on materials optimization, interface design, and scaling capabilities.^18,35^ For future development, this implies that research will center around reproducibility and reduced energy consumption.

In contrast to other review papers that tend to consider each of the above areas independently, this paper takes a more holistic approach and shows that the dependence of all the aforementioned aspects on the final characteristics of memristors can be crucial for their development into real systems for memory applications, neuromorphic processing, and intelligent computers. Further research in defect engineering, interface effects, compatible manufacturing techniques, and scalable arrays is indispensable for exploiting all the advantages of memristors in advanced VLSI and AI architectures.

## Author contributions

Robin Singla: conceptualisation, supervision, review and editing. Divya Pant: writing – original draft (Sections 2–3). Priyanshu Nautiyal: writing – original draft (Sections 4–5). Anjali Painuly: writing – original draft (Section 6), figure preparation.

## Conflicts of interest

There are no conflicts to declare.

## Data Availability

This article is a review; no primary datasets were generated. All data discussed are available in the cited references.
